# Analysis of the natively unstructured RNA/protein-recognition core in the *Escherichia coli* RNA degradosome and its interactions with regulatory RNA/Hfq complexes

**DOI:** 10.1093/nar/gkx1083

**Published:** 2017-11-09

**Authors:** Heather A Bruce, Dijun Du, Dijana Matak-Vinkovic, Katarzyna J Bandyra, R William Broadhurst, Esther Martin, Frank Sobott, Alexander V Shkumatov, Ben F Luisi

**Affiliations:** Department of Biochemistry, University of Cambridge, Tennis Court Road, Cambridge CB2 1GA, UK; Department of Chemistry, University of Cambridge, Lensfield Road, Cambridge CB2 1EW, UK; Biomolecular & Analytical Mass Spectrometry group, Department of Chemistry, University of Antwerp, 2020 Antwerp, Belgium; Astbury Centre for Structural Molecular Biology, University of Leeds, Leeds LS2 9JT, UK; School of Molecular and Cellular Biology, University of Leeds, LS2 9JT, UK; Structural Biology Brussels, Vrije Universiteit Brussel, 1050 Brussels, Belgium; VIB-VUB Center for Structural Biology, 1050 Brussels, Belgium

## Abstract

The RNA degradosome is a multi-enzyme assembly that plays a central role in the RNA metabolism of *Escherichia coli* and numerous other bacterial species including pathogens. At the core of the assembly is the endoribonuclease RNase E, one of the largest *E. coli* proteins and also one that bears the greatest region predicted to be natively unstructured. This extensive unstructured region, situated in the C-terminal half of RNase E, is punctuated with conserved short linear motifs that recruit partner proteins, direct RNA interactions, and enable association with the cytoplasmic membrane. We have structurally characterized a subassembly of the degradosome–comprising a 248-residue segment of the natively unstructured part of RNase E, the DEAD-box helicase RhlB and the glycolytic enzyme enolase, and provide evidence that it serves as a flexible recognition centre that can co-recruit small regulatory RNA and the RNA chaperone Hfq. Our results support a model in which the degradosome captures substrates and regulatory RNAs through the recognition centre, facilitates pairing to cognate transcripts and presents the target to the ribonuclease active sites of the greater assembly for cooperative degradation or processing.

## INTRODUCTION

**Figure 1.**
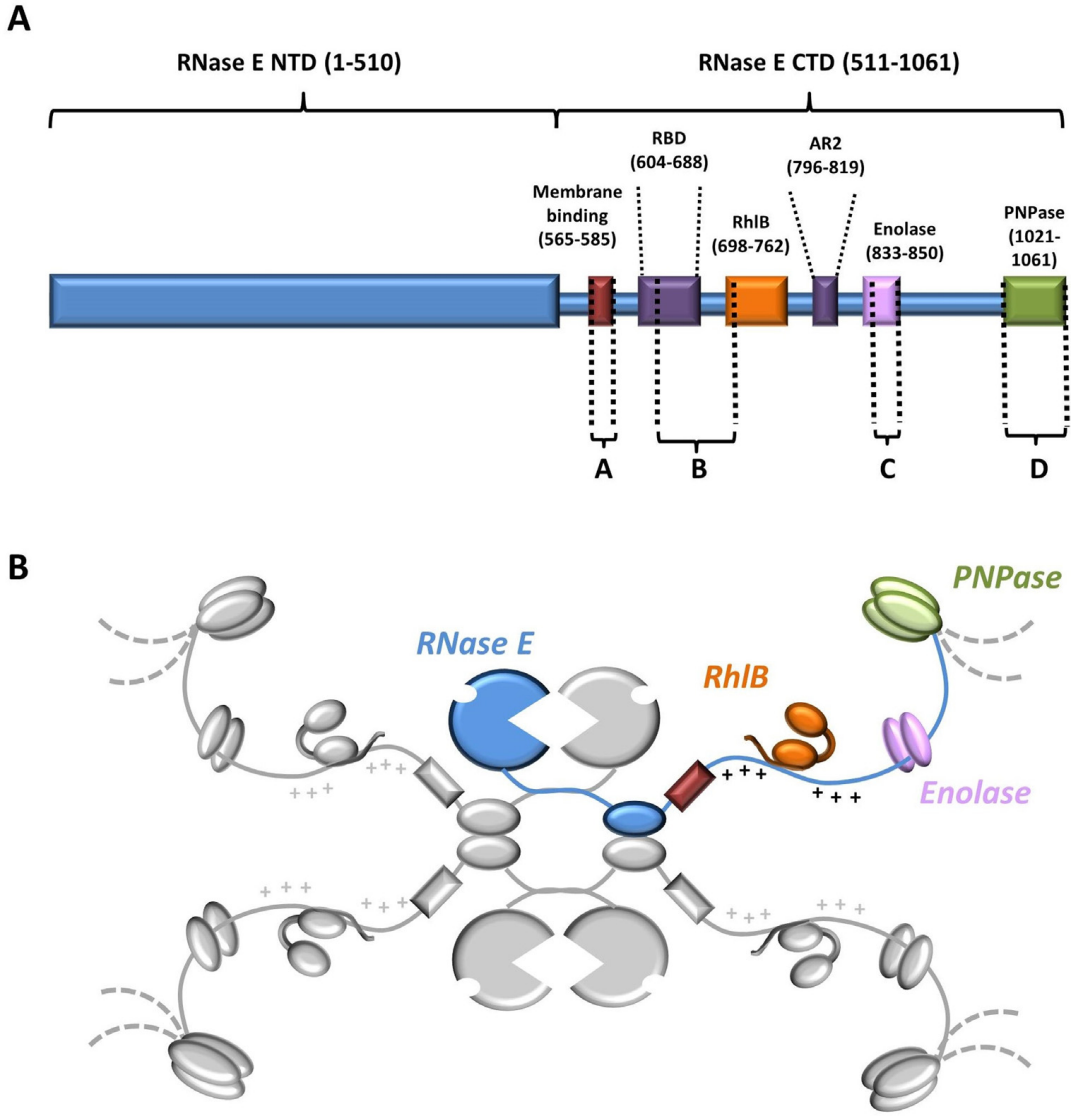
Schematic representation of the *Escherichia coli* RNA degradosome. (**A**) Linear representation of RNase E with relative positions of the RNA-, protein- and membrane binding domains. Regions indicated with letters (Site A–D) have structural propensity as predicted by bioinformatics and biophysical analyses (14–16,56). (**B**) Cartoon of the degradosome with the tetrameric RNase E as the scaffold (one protomer in blue and the others in gray). The canonical components of the degradosome: RhlB (orange), enolase (pink) and PNPase (green) are also shown in their relative positions on the C-terminal domain of RNase E. The membrane binding domain is shown in red. The arginine-rich RNA binding domains RBD and AR2 flanking the RhlB-binding site are marked with + due to their high density of positively charged residues.

From the simplest organism to the most complex, RNA contributes to the intricate regulatory processes that are mounted in response to stress, environmental changes or as part of intricate programs of development. Throughout all domains of life, regulatory RNA can affect the expression of genetic information by modulating the rates of messenger RNA translation and decay (1,2). In the model bacteria *Escherichia coli* and *Bacillus subtilis*, RNA-based regulation (riboregulation) is mediated by ribonucleases that function also in general RNA turnover and processing of precursors into mature forms (3,4). For *E. coli*, the key enzyme of RNA metabolism and riboregulation is RNase E, a conserved endoribonuclease which forms a large multi-enzyme complex, known as the degradosome, that incorporates the DEAD-box helicase RhlB, the phosphorylytic exoribonuclease polynucleotide phosphorylase (PNPase) and the glycolytic enzyme enolase (5) (Figure 1). Auxiliary proteins, such as poly-A polymerase, are also recruited in sub-stoichiometric amounts depending on growth conditions (6) and may tailor and direct the activity of the degradosome. *In situ* cross-linking followed by RNA deep sequencing have cataloged hundreds of small regulatory RNAs (sRNAs) and mRNAs that are associated with RNase E (7). The action of sRNAs in *E. coli* and numerous bacterial species is facilitated by RNA chaperones such as Hfq, which is a member of the widely occurring Sm/Lsm protein family (8,9). Hfq cooperates with RNase E to activate the cleavage of target RNAs that are tagged by cognate sRNAs (10,11).

The N-terminal half of RNase E is highly conserved and confers the degradosome with hydrolytic endoribonuclease activity. Crystal structures of this domain indicate how the enzyme recognizes and cleaves single-stranded transcripts and how it can be activated by the 5′ monophosphate group of substrate RNAs (12,13). The C-terminal half of the enzyme, which is considerably more variable in molecular evolution compared to the N-terminal half, provides the scaffold of the degradosome assembly. This scaffolding region is predicted to be predominantly natively disordered, with small linear recognition motifs that interact with the canonical protein components, RNA and the cytoplasmic membrane that consequently compartmentalizes the assembly to the periphery of the cytoplasm (14–18). The organization of the degradosome enables a high degree of component cooperation. For example, the DEAD-box helicase RhlB aids PNPase processivity by remodeling or unwinding substrates (19,20). Thus, the presence of RhlB ensures that RNA secondary structures are disrupted or protein–RNA interactions are remodeled to improve degradation efficiency.

Interplay between the degradosome components also contributes to substrate recognition and sRNA-mediated regulation. For instance, ablation of RNase E’s scaffolding C-terminal domain diminishes the efficiency of co-degradation of the sRNA–mRNA pair, RyhB–*sodB* (21,22). These results imply that the degradosome might be important for presenting the RNA duplex to the catalytic domain of RNase E. Disruption of the degradosome assembly also slows substrate cleavage rates (23), as shown quantitatively by single molecule studies of the SgrS–*ptsG* RNA pair *in vivo* (24). Moreover, an indication of the interplay between the N- and C-terminal domains is seen with the synthetic lethality phenotype that results from combining truncation of the C-terminal domain of RNase E with mutations in the catalytic domain that impede its ability to sense the 5′ end of substrates (25).

A key feature of the C-terminal portion of RNase E is its interaction with the RNA chaperone Hfq (23,26) with potential ramifications for sRNA activity. Generally, studies on the *rpoS* mRNA have suggested that Hfq-binding of target mRNA proximal to the site of sRNA pairing is crucial for regulation (27). In addition, *in vivo* crosslinking experiments have shown that Hfq preferentially binds to the 5′ side of sRNA-target sites in mRNAs and 3′ to seed sequences in sRNAs (8). The simultaneous binding of both the sRNA and cognate mRNA by Hfq may then accelerate RNA duplex formation at the rim of the protein (28) and displacement of the matching sRNA–mRNA pair mediated by the natively disordered C-terminal tail of Hfq (29). Its interaction with the degradosome may enable Hfq to cooperate with the RNA-binding functionalities of the C-terminal domain in RNase E.

Although the amino acid sequences of the C-terminal domain vary tremendously in RNase E homologs, its predicted natively disordered character is highly conserved, indicating a key biological importance for this feature (14). The RNase E C-terminal domain includes an RNA recognition core region that is predicted to be natively unstructured but is critical for functionality (23, 30–33) and in facilitating the degradation of structured transcripts by PNPase (19,32). The key regulatory role of this region is emphasized by the finding that the corresponding core region in RNase E of the pathogen *Pseudomonas aeruginosa* is targeted by a phage protein to re-program RNA degradation activity (34). Like the natively disordered regions in other proteins, this core region may interact with cognate partners in a highly specific manner through folding-mediated recognition (35) or modulate binding without folding via transient interactions (36). However, studying unstructured domains such as those occurring in the degradosome is not feasible with conventional crystallographic approaches alone. To gain insight into the scaffolding domain of the degradosome, we have used a combination of biophysical approaches to study the solution properties and functions of an RNA recognition core within the C-terminal region. Our results reveal that this core recruits sRNA–Hfq binary complexes to form ensembles of semi-compacted conformational states. We propose how such effector assemblies can facilitate mRNA target recognition and handover to the catalytic centres of the degradosome assembly in a highly dynamic manner.

## MATERIALS AND METHODS

### Preparation of the RNA degradosome recognition core region

*Escherichia coli* BL21 (DE3) was transformed with pRSFDuet-1-(*RNase E 603–850-hexahistidine tag and RhlB)*, with and without pET11-a-(*Enolase)*, for the RNase E 603–850/RhlB/enolase ternary complex and RNase E 603–850/RhlB binary complex respectively. Liquid cultures were grown in LB medium, at 37°C with appropriate antibiotics (50 μg/ml kanamycin and 100 μg/ml carbenicillin). At OD600nm 0.5, expression was induced with 1 mM isopropyl 1-thio-β-D-galactopyranoside (IPTG) and the culture incubated at 18°C overnight. Cells were harvested by centrifugation and the cell pellet re-suspended in lysis buffer (50 mM Tris–HCl pH 7.5, 250 mM KCl, 500 mM NaCl, 10 mM MgCl_2_, 5 mM β-mercaptoethanol and 10% (v/v) glycerol, protease inhibitor cocktail tablet (Roche)). Cells were lysed using Emulsiflex-05 (Avestin), and the clarified lysate transferred onto a HiTrap chelating HP column (GE Healthcare) pre-equilibrated with 50 mM Tris–HCl pH 7.5, 500 mM NaCl, 250 mM KCl, 10 mM MgCl_2_ and 5 mM β-mercaptoethanol (Buffer A). To remove contaminating nucleic acid, ∼30 ml of Buffer A with 100 mM Urea was passed through the column followed by re-equilibration with Buffer A. The protein was eluted at 4°C with a linear gradient of Buffer B (Buffer A plus 300 mM imidazole). Fractions containing all three components of the complex were pooled and dialyzed against 50 mM Tris–HCl pH 7.5, 200 mM NaCl, 200 mM KCl, 10 mM MgCl_2_ and 10 mM dithiothreitol (DTT) (Buffer C), and then loaded onto a HiTrap Heparin HP column (GE Healthcare) equilibrated with Buffer C, all at 4°C. The column was washed with Buffer C and the protein was subsequently eluted with a linear gradient of 0–35% Buffer D (Buffer C with 2 M NaCl). The purest fractions were loaded onto a Superdex 200 gel filtration column (GE Healthcare) equilibrated with 50 mM Tris–HCl pH 7.5, 200 mM NaCl, 200 mM KCl, 10 mM MgCl_2_, 10 mM DTT and 5% (v/v) glycerol (Buffer E), and after analysis by sodium dodecylsulphate polyacrylamide gel electrophoresis (SDS-PAGE), selected fractions were pooled, concentrated and flash frozen in liquid nitrogen and stored at −80°C.

### RNase E 603–850

RNase E 603–850 with a C-terminal hexahistidine tag was prepared by overexpressing all three components that make up the RNase E 603–850/RhlB/enolase ternary complex in *E. coli*, as above, to confer protection to the RNase E peptide from cellular proteases. At the metal-affinity chromatography stage, RhlB and enolase were removed from RNase E 603–850 by washing the column with 50 mM Tris–HCl pH 7.5, 0.2 M KCl, 0.4 M NaCl, 8 M Urea, 10 mM MgCl_2_ and 5 mM B mercaptoethanol (Buffer F). The column was re-equilibrated with Buffer A and RNase E 603–850 eluted with a gradient of Buffer B, as above. After SDS-PAGE analysis, fractions containing only RNase E 603–850 were pooled, dialysed against Buffer E, concentrated, flash frozen and stored at −80°C.

### Preparation of Hfq and MicC

These were prepared as described in (11).

### Size-exclusion chromatography coupled to multi-angle light scattering (SEC-MALS)

Samples of RNase E 603–850/RhlB/enolase ternary complex and RNase E 603–850/RhlB binary complex, at 5.6 and 4.0 mg/ml respectively (120 μl each), were loaded onto a Superdex 200 10/300 gel filtration column (GE Healthcare) equilibrated with 50 mM Tris–HCl pH 7.5, 100 mM NaCl, 50 mM KCl, 5 mM MgCl_2_ and 2 mM DTT). The output flow was connected to a light detector (DAWN 8^+^; Wyatt technology) using a wavelength of 664 nm with eight fixed angle detectors, in addition to a refractive index detector (OptiLab T-Rex; Wyatt technology) with a wavelength of 658 nm. Data analysis was carried out using the ASTRA 6 software package (Wyatt technology), using the refractive index of the buffer as a baseline and the refractive index increment for protein *dn/dc* = 0.185 ml/g.

### Small-angle X-ray scattering (SAXS) data collection and analysis

Small-angle X-ray scattering (SAXS) data were collected on the SWING beamline at Soleil synchrotron (Gif-sur-Yvette, France). Samples were stored at 288 K in a robotic sample chamber and automatically loaded onto a Superdex 200 Increase 3.2/300 gel-filtration column (GE Healthcare) equilibrated with 50 mM Tris–HCl pH 7.5, 100 mM NaCl, 100 mM KCl, 10 mM MgCl_2_, 10 mM DTT and 5% glycerol (v/v), at a flow rate of 150 μl min^−1^ by a High performance liquid chromatography (HPLC) instrument (Agilent), directly before elution into the sample detection chamber, where a monochromatic beam illuminated the sample with a wavelength of 1.022 Å as it flowed through. The sample-detector distance was 1.79 m. During the elution, 250 scattering measurements were taken with 1.5-s time-frames and 0.5-s dead-time between frames. The in-house program *FOXTROT* (37) was used to normalize and radially average the data. After averaging 20–30 buffer frames in *PRIMUS* (38), the program *DATASW* (39) was employed to (i) subtract the buffer average from each sample frame and (ii) calculation of invariants (*I*_0_, *R*_g_, molecular weights (*M_w_*)). For each sample, an elution profile was generated with the *I*_0_/*R*_g_ variation plotted versus recorded frame number. For RNase E 603–850/RhlB/enolase ternary complex, RNase E 603–850/RhlB binary complex and RNase E 603–850 alone, a region was selected by *DATASW* and averaged to generate the final scattering curve used for subsequent analysis.

The *ATSAS* package (40) was used to analyze the data. The scattering curves were initially viewed in *PRIMUS* to check for any aggregation or inter-particle interference which can manifest itself in an upward or downward turn of the curve at low *q-v*alues, respectively. For the ternary complex, binary complex and RNase E 603–850 samples, the *R*_g_ was obtained from the slope of the Guinier plot in *PRIMUS* within the region defined by *q*_min_*< q < q*_max_*w*here *q*_max_ < 1.3/*R*_g_ and *q*_min_ is the lowest angle data point that the program finds acceptable to include. For each sample, the data were transformed from reciprocal to real space using the indirect transform program *GNOM* (41) which generates a distribution of the intra-atomic distances (r) in a particle, P(r) function. The maximum distance (*D*_max_) was selected by permitting the P(r) curve to run smoothly to zero. The *R*_g_ was also estimated from the P(r) function, which, unlike *R*_g_ estimation using Guinier region, takes the whole scattering curve into account and hence for flexible systems is more reliable estimate of invariants (42). The excluded volume was estimated using *DAMMIN* in P1 and P(r) function, generated from the data truncated to 0.25 Å^−1^. For *GASBOR* reconstructions, P(r) function was generated from the data truncated at high angles to remove very noisy parts. A total of 10 independent reconstructions were performed, and the models were averaged with the program *DAMAVER* (43), which provides a value of normalized spatial discrepancy (NSD) representing a measure of similarity among different models. NSD values close to one indicate that the models have very similar shapes. Figures were created in the program *CHIMERA* (44).

For flexibility assessment of RNase E 603–850, a random pool of structures was generated using the *EOM* suite (45,46) with default parameters (‘Random-coil’ chain type), resulting in a random pool of 10 000 C-α trace models of RNase E 603–850. Next, a computational pipeline *FULCHER* (Shkumatov A. *et al*, unpublished data) was used to convert C-α trace models to all-atom models, with subsequent model validation using the *MOLPROBITY* clash score of 40, resulting in 3770 models in the random pool. Finally, the genetic algorithm *GAJOE* was run 10× to obtain an ensemble of models that best describes the experimental SAXS data.

### Hydrogen-deuterium exchange analyses of complexes

Samples of RhlB, RNase E 603–850 and the RNase E 603–850/RhlB binary complex were diluted in labeling buffer (10 mM phosphate D_2_O, pD 7.0) and deuterated for various times (0, 30, 60, 300, 900, 1200, 2700, 3600, 5400 and 10800 s) followed by reaction quenching in Q buffer (100 mM phosphate H_2_O, pH 2.66), prior to injection onto a pepsin column (porozyme immobilized pepsin cartridge) at a flow rate of 100 μl/min, all at 20°C. Peptides were separated on a BEH C18 column (1.7 μm, 1.0 × 100 mm) using nanoACQUITY UPLC (Waters) and analyzed on a Synapt G2-Si HDMS, at 0°C. DynamXTM v 3.0 software (Waters) was used for the automatic data processing to generate deuterium uptake curves for all the detected peptides. The amount of deuterium in each peptide was determined by measuring the centroid of the isotopic distribution. All of the data were derived from at least two independent experiments.

### Co-expression, purification and crystallography of the enolase/AR2–EBS complex

The *rne*_2346–2550_ and enolase genes were cloned into pETDuet vector, resulting in a construct co-expressing enolase and an N-terminal hexahistidine-tagged AR2-enolase binding site (EBS) corresponding to residues 782–850 of RNase E. The construct was transformed into *E. coli* strain Rosetta (DE3). Cells were grown at 37°C until the culture reached an absorbance at 600 nm of 0.5–0.6 and was then induced by the addition of 0.3 mM IPTG at 25°C for 3 h. The cells were harvested by centrifugation, resuspended in 50 ml of lysis buffer (20 mM Tris pH: 8.0, 400 mM NaCl, 40 mM KCl, 1 mM MgCl_2_, 5 units/ml DNase I and 1 tablet/50 ml protease inhibitor mixture tablet), and lysed using a high pressure homogenizer (Emulsiflex) at 15 000 Psi. Cellular debris was removed by centrifugation and imidazole was added to the supernatant to a final concentration of 10 mM. Hexahistidine tagged AR2–EBS/enolase complex was purified by affinity chromatography using a HiTrap chelating column (GE Healthcare) equilibrated with wash buffer (20 mM Tris pH 8.0, 400 mM NaCl, 40 mM KCl and 1 mM MgCl_2_). The column was washed with 30 mM imidazole added to wash buffer and GF buffer (20 mM Tris pH 8.0, 150 mM NaCl and 5 mM MgCl_2_), respectively. Purified AR2–EBS/enolase complex was eluted with 300 mM imidazole in GF buffer, concentrated and loaded onto a Superdex 200 column equilibrated with GF buffer. Fractions containing purified AR2–EBS/enolase complex were pooled and concentrated to 65.6 mg/ml.

Crystals were grown at 18°C using the hanging-droplet vapor diffusion method by mixing 200 nl of AR2–EBS/enolase complex with 200 nl of reservoir solution (0.2 M potassium thiocyanate, 0.1 M Bis-Tris propane (pH 8.5) and 20% (w/v) PEG 3350)). Crystals appeared several hours after setting-up the crystallization trial and reached the final size in 1 week. The crystals were transferred briefly into reservoir solution supplemented with 25% PEG 400 as cryoprotectant before flash-freezing in liquid nitrogen. X-ray diffraction data were collected at 100 K from cryoprotected crystals at beamline I04 at the Diamond Light Source (Didcot, UK). A complete dataset of AR2–peptide C/enolase complex crystals was collected to a resolution of 2.0 Å (Table 1). The data were processed and scaled using HKL2000 and SCALEPACK, respectively. Molecular replacement was performed using the CCP4 suite program Phaser. The structure of the *E. coli* enolase dimer in complex with the minimal binding segment of RNase E (PDB entry: 2FYM) was used as the search model to construct an initial model of AR2–EBS/enolase complex. This initial model was refined using Refmac5. The residues outside of the electron density map in the refined model were then deleted. The non-crystallography symmetry (NCS) operators were obtained from this partial model and used to carry out density modification with the CCP4 suite program Parrot. The missing residues of one protomer were manually built into the modified electron density map using Coot and directly placed in the remaining protomers based on NCS. The model was refined again with Translation/Libration/Screw (TLS), NCS and restrained refinement using Refmac5. 3-phosphoglycerate and Mg^2+^ were finally built into the electron density map manually using Coot. A summary of the crystallographic data and refinement are shown in Table 1. The program PYMOL was used to prepare figures (48).

**Table 1.** Crystallographic data collection and refinement statistics for enolase in complex with RNase E 796–850

Space group	P2_1_
Cell dimensions (Å)	*a* = 74.683, *b* = 116.923, *c* = 107.776
	*β* = 105.429
No. of observations	428987
No. of unique observations	113106
Resolution (Å)	2.00 (2.07–2.00)
Completeness (%)	98.59
Multiplicity	3.7
CC_1/2_	0.999
Rmerge	0.137 (0.439)
I/SigmaI	9.2
Refinement	
R (working set)	0.161
R free	0.197
RMSD bond lengths (Å)	0.008
RMSD bond angles (°)	1.097
Ramachandran favored (%)	97.3
Ramachandran preferred (%)	2.5
Ramachandran outliers (%)	0.3

### Investigating the interaction between enolase ±AR2–EBS and tRNA^Phe^

Samples of *E. coli* tRNA^phe^, and enolase ±AR2–EBS, at 2 μM in GF buffer were prepared. The RNA was heated at 50°C for 2 min and were cooled to room temperature for 5–10 min. Two microliter of tRNA^phe^ was mixed with 0, 1, 2 and 4 μl of enolase ±AR2–EBS. GF buffer was added to each sample to a final volume of 10 μl. The samples were incubated at 30°C for 30 min, and then transferred to ice. A total of 4 μl of ice-cold 5% sucrose was added to each sample and the mixtures were loaded on 1% (w/v) agarose gel and run at 100 V at 4 °C in 0.5× TBE running buffer. The gel was stained with SYBR gold (Invitrogen) and visualized with a ultraviolet (UV) imager.

### Characterization of the recognition core interaction with MicC and Hfq by native polyacrylamide gel electrophoresis (PAGE) followed by LC-MS/MS analysis

Samples of the recognition core, Hfq and recognition core + Hfq, were all incubated in the presence and absence of MicC, in equimolar ratios, at a final concentration of 250 nm (10 μl final volume) in the interaction buffer: 50 mM Tris–HCl pH 7.5, 100 mM NaCl, 50 mM KCl, 10 mM MgCl_2_ and 5 mM DTT, for 5–10 min at 4°C. Following the addition of 5 μl loading buffer (interaction buffer with 50% (v/v) glycerol), each sample was analyzed by an 8% native polyacrylamide gel (200 mM Tris–HCl pH 8.5, acrylamide: bisacrylamide 37.5:1 and 10% (v/v) glycerol) run in 1× Tris glycine at 150 V for 1 h at 4°C. The gel was stained with SYBR Gold (Invitrogen) and visualized with a UV imager. The shifted species corresponding to the recognition core-MicC and recognition core-MicC-Hfq were excised. The corresponding migration positions in the gel for protein only controls for the two complexes were also excised. All four bands were analyzed by LC-MS/MS (carried out by Dr Mike Deery at the Cambridge centre for proteomics). The experiment was repeated 5× for the recognition core-MicC and recognition core-MicC-Hfq bands in addition to the recognition core-MicC-Hfq (protein only control) band, and 4× for the recognition core-MicC (protein only control) band.

The exponentially modified protein abundance index (emPAI) is a measure for absolute protein abundance after mass spectrometry analysis (49). The emPAI was determined for each identified component in each sample using the following equation: *emPAI* = 10*^PAI^* – 1, where *PAI* is the number of observed peptides divided by the number of observable peptides per protein. The emPAI values were averaged over the different experiments, with their standard deviations calculated. The emPAI values for the protein only controls were subtracted from the values for the putative complexes, resulting in a final background corrected recognition core-MicC and recognition core-MicC-Hfq species, with their calculated cumulative errors.

## RESULTS

### Properties of an RNA/protein recognition core within the RNA degradosome

**Figure 2.**
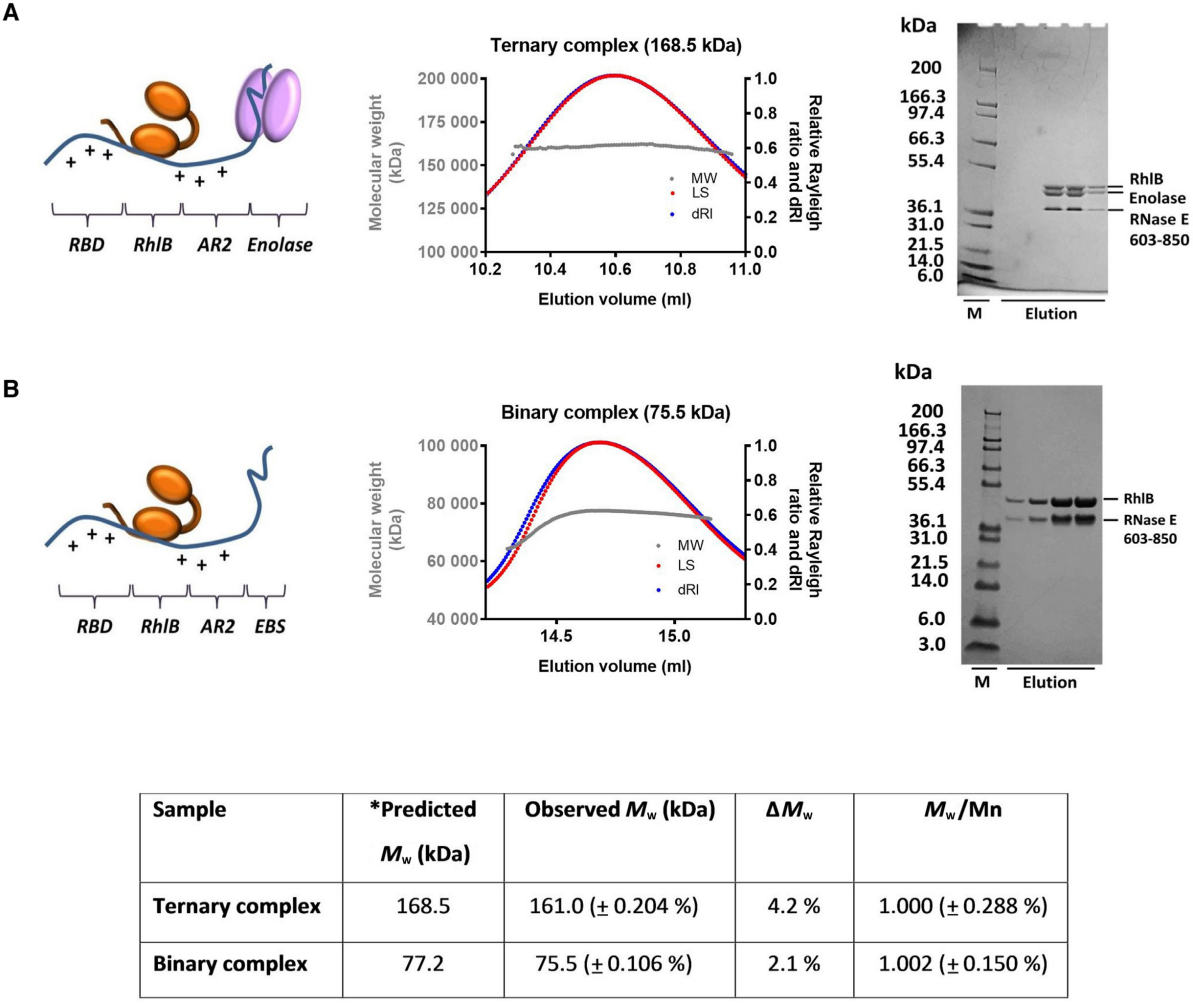
Subunit composition of subassemblies of the RNA degradosome. The left panels show cartoon schematics of the subassemblies (ternary and binary complexes); the central panels show analysis by SEC-MALS in which LS (red) and differential refractive index (dRI, blue) are plotted in addition to *M*_w_ (gray); the right panels present SDS-PAGE images for the peak fractions. (**A**) The portion of RNase E encompassing residues 603–850 (blue) (with C-terminal Hexa-histidine tag; hereafter RNase E 603–850) interacts with RhlB (orange) and enolase (pink) to form a stable ternary complex composed RNase E 603–850: enolase: RhlB in a 1:2:1 molar ratio as indicated by the predicted and observed *M*_w_. (**B**) RNase E 603–850 and RhlB form a stable binary assembly of 1:1 RNase E 603–850: RhlB. Predicted and observed *M*_w_ of the ternary and binary complexes, with their polydispersity values are shown in the table (inset). Note: RNase E 603–850 migrates slightly slower than expected, possibly due to highly charged regions or high proline content within the peptide (14,56).

A segment of the *E. coli* RNase E C-terminal domain, encompassing the binding sites for enolase and RhlB (corresponding to residues 603–850; hereafter RNase E 603–850), also bears two arginine-rich RNA binding domains, which are referred to as the RNA-binding domain (RBD) and AR2 (arginine-rich region 2) (30–32) (Figure 1). This region in the degradosome has been implicated in the efficient sRNA-induced degradation of target mRNAs by RNase E (23,33,50) and in facilitating the degradation of structured transcripts by PNPase (19,32). To identify the subunit stoichiometry of the recognition core region, subassemblies comprising RNase E 603–850 and RhlB, with and without enolase, were co-expressed, purified and analyzed by size-exclusion chromatography coupled with multi-angle light scattering (SEC-MALS) (Figure 2). The observed *M*_w_ are indicative of a stable and discrete assembly for the RNase E 603–850: enolase: RhlB ‘ternary complex’ and the RNase E 603–850: RhlB ‘binary complex’, with subunit ratios of 1:2:1 and 1:1, respectively. The polydispersity values for the ternary and binary complexes (Figure 2; inset table) indicate that both samples are monodisperse. Dynamic light scattering (DLS) measurements for the complexes also support monodisperse systems (Supplementary Figure S1).

The component stoichiometries of the ternary and binary complexes determined by SEC-MALS are consistent with data from native electrospray ionization (ESI) mass spectrometry (Supplementary Figure S2). In addition to the complexes, the individual components (RNase E 603–850, RhIB and enolase dimer) were observed in the spectrum (Supplementary Figure S2A). The measured masses are all in close agreement with predicted *M*_w_. The native ESI mass spectrometry analysis also reveals that RNase E 603–850 has a broad charge distribution in the spectra, which indicates that it has conformational disordered character. While the other subunits show compact charge state distributions with typically 3–4 charge states visible, RNase E 603–850 shows a distribution spanning 44+ to 24+. This unusually high number of charges for an ∼30 kDa protein whereby many sites are accessible for protonation indicates that RNase E 603–850 lacks a well-defined conformation, supporting the earlier bioinformatic predictions for this region (15). Ion-mobility mass spectrometry was also used to analyze the subassemblies. The arrival time of each particle through the traveling-wave ion mobility cell can be extracted from this plot and converted into a collision cross section value (Supplementary Figure S2B and C). For the most native-like charge states, the estimated collision cross sections for the ternary and binary complexes were 8250.6 and 4792.1 Å^2^, respectively. By contrast, the collision cross section value for RNase E 603–850 (6741.9 Å^2^) is much greater than predicted for a globular protein of corresponding weight, indicating that this particle has significant conformational variation (Supplementary Figure S2D).

**Figure 3.**
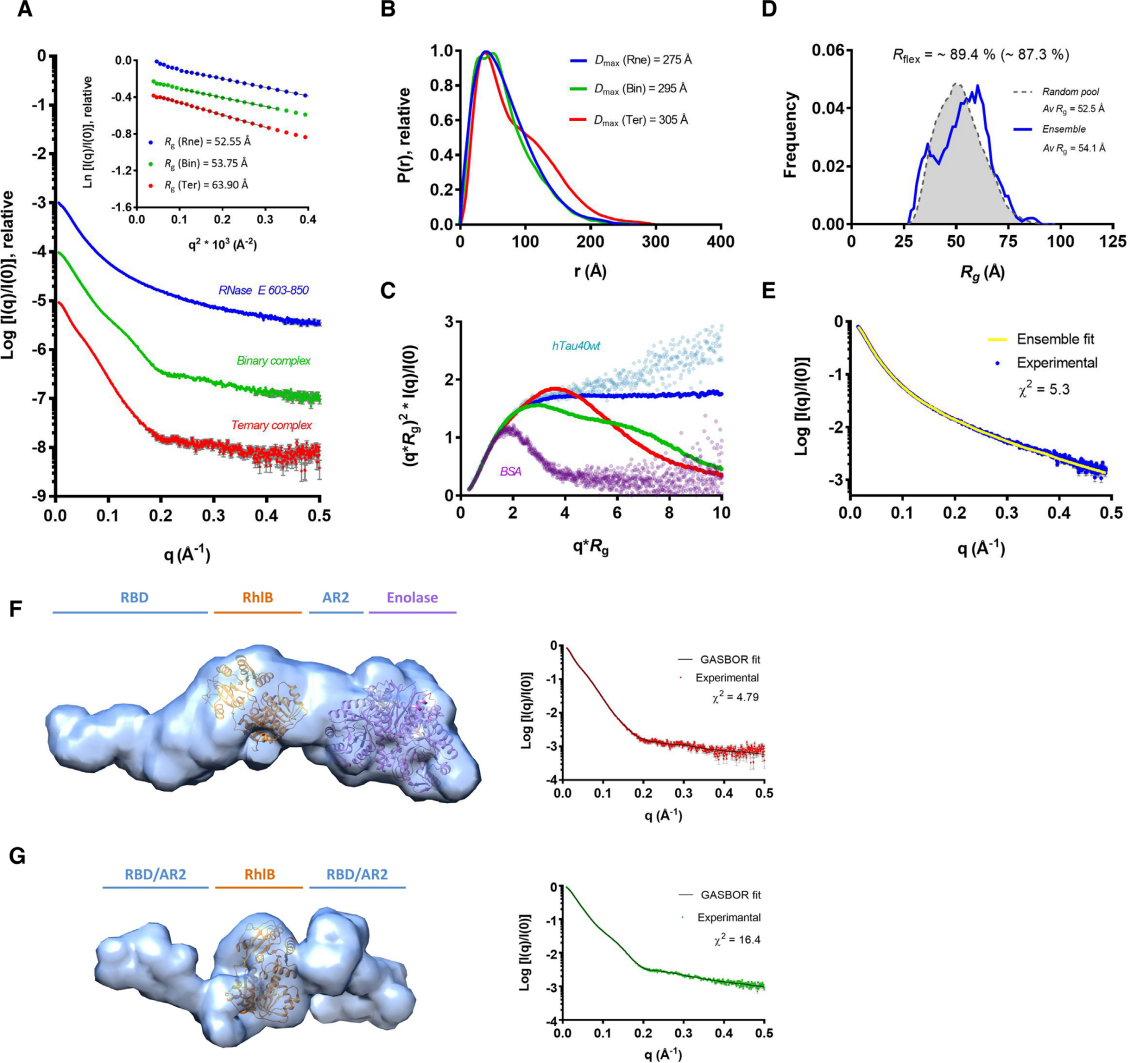
SAXS analysis of degradosome subassemblies; RNase E 603–850/RhlB/enolase ternary complex (Ter, red), RNase E 603–850/RhlB binary complex (Bin, green) and RNase E 603–850 (Rne, blue). (**A**) Scattering intensity profiles with inset Guinier regions incorporating fits and derived *R*_g_ values. (**B**) P(r) distribution functions, with respective *D*_max_ values. (**C**) Dimensionless Kratky plot overlay of the ternary complex, binary complex and RNase E 603–850 compared to references; the globular BSA and highly flexible hTau40wt proteins (66) (**D**) Flexibility assessment of RNase E 603–850: the *R*_g_ distribution for the random pool of models (gray) and for the ensemble of conformations that altogether give a good fit to the scattering intensity curve (blue) are shown, with *R*_flex_ values of 87.3 and 89.4%, respectively. (**E**) RNase E 603–850 experimental scattering intensity curve (blue) with ensemble model fit (yellow line). (**F** and **G**) *Ab initio* reconstruction of the degradosome recognition core. Molecular envelopes of (F) the RNase E 603–850/RhlB/enolase ternary complex and (**G**) RNase E 603–850/RhlB binary complex, calculated in GASBOR. A homology model of RhlB, generated in Phyre^2^ program (67) based on the crystal structure of the *Drosophila* DEAD box helicase VASA (51), in addition to the crystal structure of enolase (PDB: 3H8A; 52) were docked into the shapes using the automatic function in the program Chimera (44). The space flanking RhlB corresponds to the RBD and AR2, for which there are no experimentally determined structural models. The X-ray scattering intensity profile of the ternary complex (red) and binary complex (green) overlaid on respective theoretical curves from *ab initio* models (black line) are shown to the right of the respective models. Visually the experimental scattering intensities have an excellent fit to the model scattering intensity curves, although χ^2^ values are relatively high (4.8 and 16.4, respectively) due to high signal-to-noise ratio.

To further investigate the overall structural conformation and degree of flexibility of the subassemblies in solution, SAXS was employed (Figure 3). The Guinier derived radius of gyration (*R*_g_) values for RNase E 603–850, the binary complex and the ternary complex are ∼53, ∼54 and ∼64 Å, respectively (Supplementary Table S1). Comparing these values with expectations for globular proteins of the corresponding *M*_w_ suggests that all three particles have non-globular shapes. In contrast, the computationally predicted *R*_g_ values for a homology model of RhlB based on the structure of VASA helicase from *Drosophila* (32,51) and the crystal structure of enolase (52) are in accord with the expected values for globular proteins (53) (Supplementary Figure S4A).

The comparatively featureless scattering intensity curve and the smooth and extended P(r) distribution function of RNase E 603–850 indicate that the protein is highly flexible in solution (Figure 3A and B, respectively). Moreover, the dimensionless Kratky profile (dKratky) (54) of this RNase E segment is characteristic of an intrinsically disordered protein (IDP), with the intensity increasing gradually and the absence of a peak at lower angles (Figure 3C). The conformational variability of RNase E 603–850 was probed using the ensemble optimization method (EOM) (45) and all-atom modeling (Figure 3D), followed by model validation using the Molprobity score. Model selection based on the experimental SAXS data indicate that the latter could be represented by an ensemble of 14 conformations, providing an excellent fit to the experimental scattering curve (Figure 3E and Supplementary Figure S4). The calculated distribution of *R_g_* values from the ensemble samples a similar space to that of the random pool, suggesting that the isolated RNase E 603–850, in the absence of its helicase and enolase partners, is indeed highly flexible (*R*_flex_ values of the ensemble and random pool are very close at 89.4% and 87.3%, respectively). Interestingly, the average *R*_g_ value of the ensemble distribution is slightly higher than that of the random pool. Furthermore, the experimental *R*_g_ of RNase E 603–850 is greater than the value of ∼46 Å predicted for a typical IDP of the same length (45,55), indicating that the construct is not only highly flexible but also favors extended conformations in solution. The enrichment in basic residues and high isoelectric point (estimated pI 10.67) of RNase E 603–850 (14) may confer partial backbone rigidity contributing to extended conformations in solution.

RhlB binds to RNase E in the region corresponding to residues 698–762 in the degradosome assembly (56) (Figure 1). Notably, the gyration radii of RNase E 603–850 and the binary complex (comprising RNase E 603–850 and the helicase) are very close, which suggests that complex formation is accompanied with some structuring. This structuring (55) in the case of the binary and ternary complexes can be supported by appearance of additional features (bumps) in the scattering curve, small additional peak in the P(r) function and maxima in the dKratky plots (Figure 3A–C, respectively). However, both binary and ternary complexes remain extended, as supported by the long trailing tail present in the P(r) functions, in addition to their hydrodynamic radii obtained from DLS measurements which are much greater than the predicted values for globular proteins (Supplementary Figure S1).

The program *GASBOR* (57) was used to generate *ab initio* models with predefined numbers of dummy residues for binary and ternary complex. The resulting envelopes are elongated with multiple domains, though consistent with the expected volumes. The values of NSD, reflecting similarity of the independent reconstructions, are >1 for both ternary and binary complex (Supplementary Table S1), supporting the model that they have conformational heterogeneity. It should be noted that generated envelopes (Figure 3F and G) represent an average over the sets of existing conformations in solution. Nevertheless, the bulkier end of the ternary complex envelope readily accommodates a crystal structure of enolase (52), whilst a homology model of RhlB can be positioned in the central region, with space remaining either side corresponding to the RBD and AR2 regions which flank the helicase, and for which there are no high-resolution structures. Interestingly, the RBD and AR2 regions show bulkier density than expected based on their size, suggesting that these domains may not necessarily form a single static structure but are partly flexible in the complex. Taken together, the biophysical data suggest that although RNase E 603–850 may undergo some structural changes upon binding RhlB and enolase, it does not fully fold into a compact form, but instead preserves some of its dynamic character within the recognition core region of the degradosome.

### Characterising the RNase E-RhlB interaction and its implications for helicase activity and cooperation

**Figure 4.**
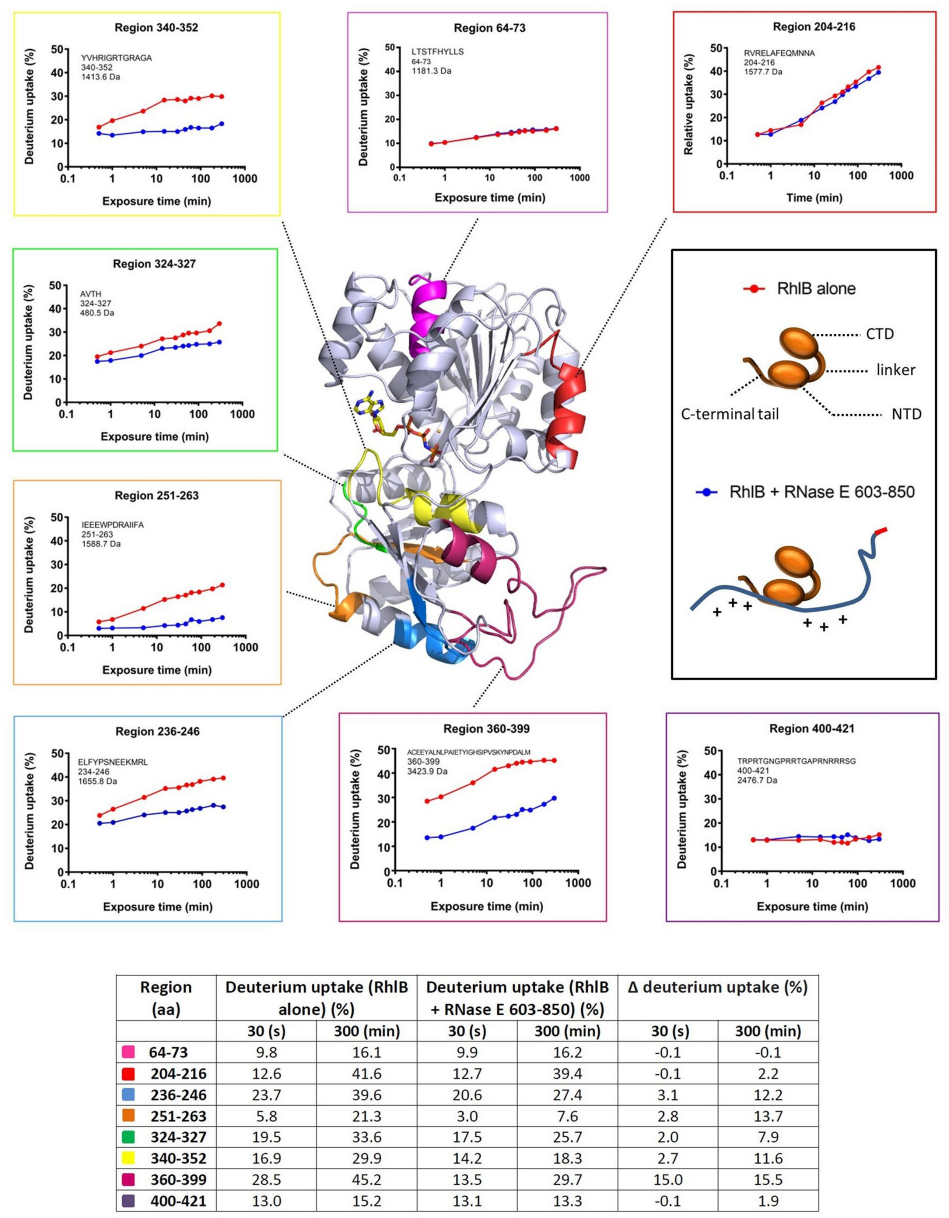
Impact of RNase E binding on RhlB exposed surfaces. The RNase E binding sites in RhlB are mapped from hydrogen-deuterium exchange analyses (HDX-MS). In the central panel is a homology model of RhlB colored in gray, with the exception of five sites in the C-terminal RecA-like domain of the helicase which have a reduced deuterium uptake when RNase E (603–850) is bound, in addition to two randomly selected sites in the N-terminal domain which do not have a different exposure to the solvent upon RNase E 603–850 binding. The C-terminal tail (region 400–421) is highly flexible and therefore not generated as part of the homology model. Non-hydrolyzable adenosinetriphosphate (ATP) shown in stick form is positioned in the catalytic site. The panels along the top, left and bottom show the hydrogen/deuterium exchange rates of selected regions in RhlB in the presence and absence of RNase E 603–850 (blue and red, respectively). The panel on the right is the cartoon schematic for the helicase domains and for the interaction mode between the helicase C-terminal RecA-like domain and RNase E. The homology model was generated with the Phyre^2^ program (67) using as a template the crystal structure of the RNA helicase VASA from *Drosophila* (51). The table (inset) provides a quantitative summary of the HDX-MS results.

In the recognition core region of the degradosome, interplay between RhlB and the flanking RBD and AR2 domains is expected to occur during the binding and remodeling of RNA (32). The ATPase and RNA unwinding activities of RhlB are boosted through its interaction with RNase E (32,58). In addition to an N- and C-terminal RecA-like domain, RhlB possesses a dynamic, positively charged C-terminal tail, which contributes to RNA binding (32,47) (Figure 4, inset schematic). It has previously been shown that RNase E binds within the C-terminal RecA-like domain of RhlB, and that the N-terminal RecA-like domain and C-terminal tail do not participate in the protein-to-protein interaction (32).

To map more precisely the RNase E interaction site on RhlB and explore how the interaction favors the activities of the helicase, we employed HDX-MS using samples of RhlB alone or in complex with RNase E 603–850 (Supplementary Figure S5). Peptide regions within RhlB that have decreased solvent exposure in the context of the RNase E 603–850/RhlB complex were mapped onto a homology model of the helicase (Figure 4). The sites of protection correspond to amino acid regions located in the C-terminal RecA-like domain of RhlB (residues 236–246, 251–263, 324–327 and 340–352 and 360–399), and do not extend to the N-terminal RecA-like domain or C-terminal tail. The C-terminal tail of RhlB was however found to have a time independent exposure to the solvent, which suggests that it is flexible, in agreement with earlier reports (32,58).

The largest region in RhlB with reduced solvent exposure upon interaction with RNase E corresponds to residues 360–399, which is found distal to the ATPase active site (colored raspberry in Figure 4). Previously, region 377–383 in RhlB was identified as a putative RNase E interaction site by bioinformatic analysis (32) and the experimental data collected here support this finding. Interestingly, the HDX-MS analysis also reveals several other regions in RhlB that are protected from the solvent in the presence of RNase E 603–850. Sites 236–246 and 251–263 in RhlB (blue and orange, respectively, Figure 4) probably correspond to a single segment that overlaps with RNA-binding motif IV, which is one of the signature sequence elements of DEAD-box helicases (Supplementary Figure S5). Interestingly, site 340–352 is an unstructured region located in close proximity to the catalytic site (yellow, Figure 4), and overlaps with a key nucleotide binding sequence in the helicase, corresponding to motif VI. In addition, region 324–327 (green, Figure 4) is found close to the terminal histidine residue of DEAD-box signature motif V at position 321, which has been previously identified as a conserved residue amongst RhlB homologs, but not in other DEAD-box helicases of *E. coli* (47). Potentially, RNase E binding to region 324–327 in RhlB alters the positioning of the peptide backbone and places the upstream His 321 in a more favorable position to support ATPase activity. In this way, RNase E binding to RhlB could impact favorably on ATP binding, hydrolysis and release.

The converse interaction site in the RNase E 603–850/RhlB complex was also investigated by HDX-MS (Supplementary Figure S6). The absence of a time dependent H/D exchange with the solvent was apparent throughout the peptide regions detected in RNase E 603–850, supporting the native mass spectrometry and SAXS observations described above which indicate that the protein is highly flexible. Of note, regions corresponding to residues 711–739 and 749–765 in RNase E 603–850 display a decrease in solvent accessibility upon RhlB binding, supporting previous studies (31,32) which locate the RhlB binding site (RBS) as residing within the region 698–762. Interestingly, a reduction in deuterium uptake in RNase E upon RhlB binding is also observed for a site within the RBD region and another site which overlaps with the AR2 region, corresponding to residues 675–685 and 774–807, respectively (Supplementary Figure S6B). This suggests that RhlB binding to RNase E 603–850 influences the structural conformation of the RNA binding domains. As the exposure in these regions is still mostly time independent upon the RhlB interaction, they are unlikely to form stable structures, but may instead sample a different range of conformations which are less open. The effects of RhlB binding to RNase E 603–850 also extend to the EBS (residues 823–850); a reduction in solvent accessibility occurs in this region as a result of complex formation.

### Enolase influences the AR2 domain

**Figure 5.**
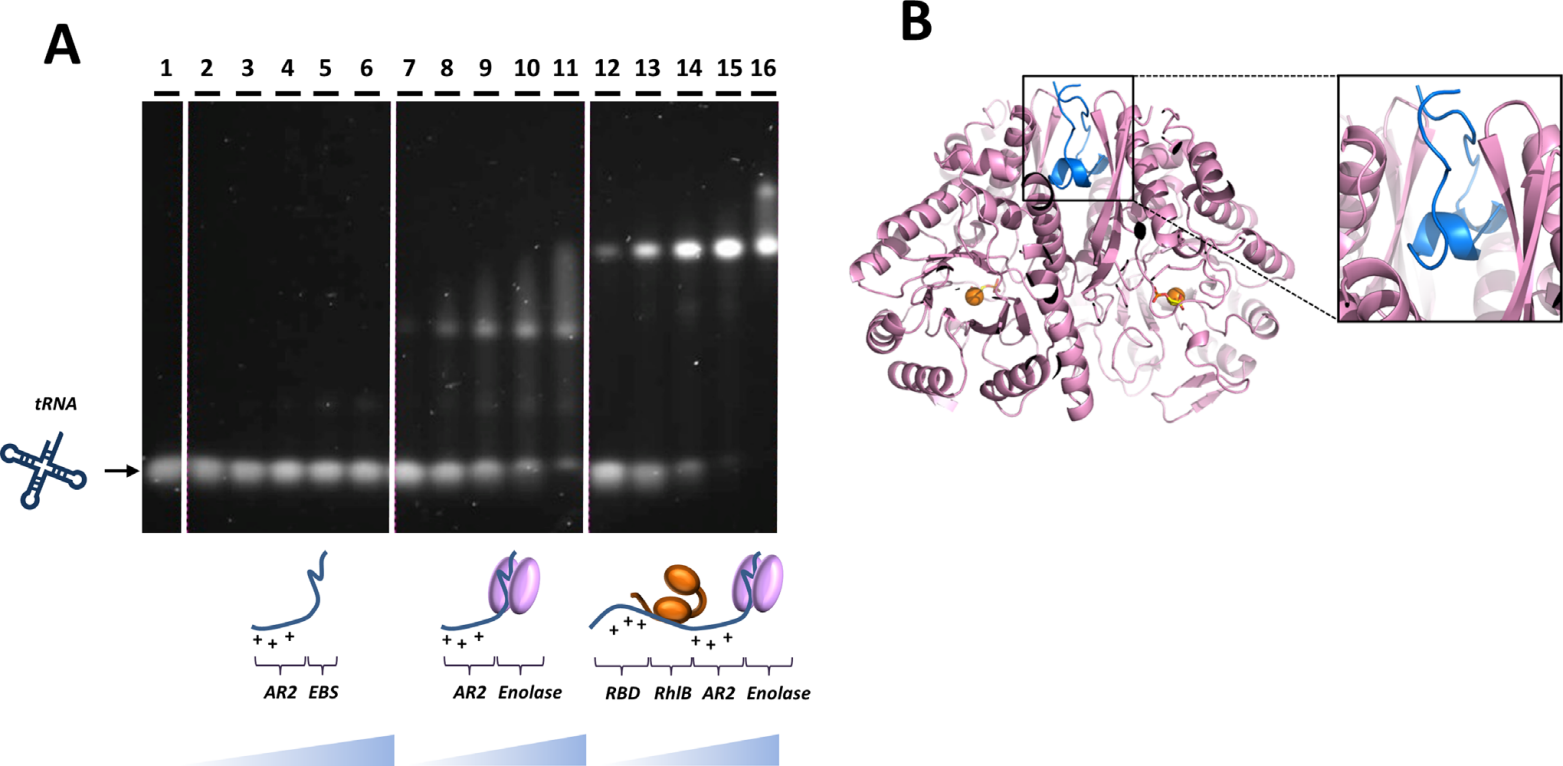
Recognition of RNase E and enolase, and its impact on the RNA binding capabilities of AR2. (**A**) Native agarose gel shift assay stained for RNA. The affinity of the AR2–EBS segment (blue) for tRNA^phe^ (dark blue) is enhanced in the presence of enolase (pink). tRNA^Phe^ (lane 1) does not readily interact with AR2–EBS region of RNase E (lanes 2–6). However, the same region of RNase E in complex with enolase does form a super-shifted species with tRNA^Phe^ (lanes 7–11). The RNase E 603–850+RhlB+enolase ternary complex also forms a shifted species with tRNA^Phe^, with a higher affinity (lanes 12–16) due to the additional presence of RhlB and RBD. (**B**) The crystal structure of the enolase in complex with RNase E segment C and AR2 (blue). The electron density for the AR2 is poorly resolved and the model shows only the EBS in the intra-protomer binding cleft.

The EBS in RNase E is highly conserved within the Vibrionales, Pasteurellales and Enterobacteriales of the γ-proteobacteria (14), suggesting that it bears functional importance. In the recognition core region of the degradosome, the sequence of the AR2 RBD (residues 796–819) is situated adjacent to the EBS (Figure 1). The AR2 region is also a conserved motif, but as mentioned earlier it is computationally predicted to lack any standard secondary structure (15). To explore the influence of enolase binding on the RNA-binding capabilities of the physically adjacent AR2 domain, a native gel shift assay was undertaken using RNA and AR2–EBS RNase E peptide, with and without enolase bound (Figure 5A). *E. coli* tRNA^Phe^ was chosen as a structured RNA substrate that RNase E would encounter in processing tRNA precursors. Interestingly, the presence of enolase facilitates the interaction between AR2 and tRNA^Phe^ (compare the first three panels; lanes 1–11). When the interaction between the entire recognition core region and tRNA^Phe^ was probed under the same conditions, the shifted species formed more readily, indicating that this assembly possesses a higher affinity for RNA, probably owing to the additional presence of RhlB and the RBD (compare lanes 7–11 and 12–16). Alone, enolase does not readily bind RNA under the tested conditions (Supplementary Figure S7), consistent with earlier findings (19).

To explore the structural impact of enolase binding on the adjacent AR2 element, the enzyme was co-crystallized in complex with the RNase E segment encompassing the EBS and AR2 regions. Well diffracting crystals were obtained and the structure solved and refined to 2.0 Å resolution (Figure 5B and Table 1). Two enolase dimers occupy the asymmetric unit. In agreement with earlier results, the segment with residues 822–849 interacts with the intra-protomer cleft of the enolase dimer (32,52). The cleft has 2-fold symmetry and the EBS can enter the cleft in one of two equivalent orientations. Notably, in one of the enolase dimers, the EBS is superimposed in those two orientations. Although some broken density could be seen for the N-terminal side of the EBS, no features could be resolved unambiguously for the AR2 segment in either of the enolase dimers of the asymmetric unit. Nonetheless, some portions of the AR2 peptide could be fitted to the density at low occupancy, but it is clear that this segment assumes multiple conformations. This suggests that the AR2 region possesses significant flexibility and does not adapt a single conformation when enolase is bound to the neighboring EBS. It may be the case that the binding of enolase to the adjoining region limits conformational variation of the AR2 region or counters auto-inhibitory states, thereby favoring its interaction with RNA.

### Investigating an interaction between the recognition core and an sRNA/Hfq complex

RNase E plays a key role in sRNA-mediated degradation of mRNA. In addition to its N-terminal catalytic domain, the recognition core region of the degradosome is essential for the degradation of the sRNA SgrS and the target *ptsG* mRNA, in a process which also requires the RNA chaperone protein Hfq (23). Although the C-terminal half of RNase E is important for the efficient degradation of this and other sRNA–mRNA pairs, the extent to which the components of the degradosome are involved—such as enolase and RhlB—has not yet been fully established (22,26,33,47,59).

**Figure 6.**
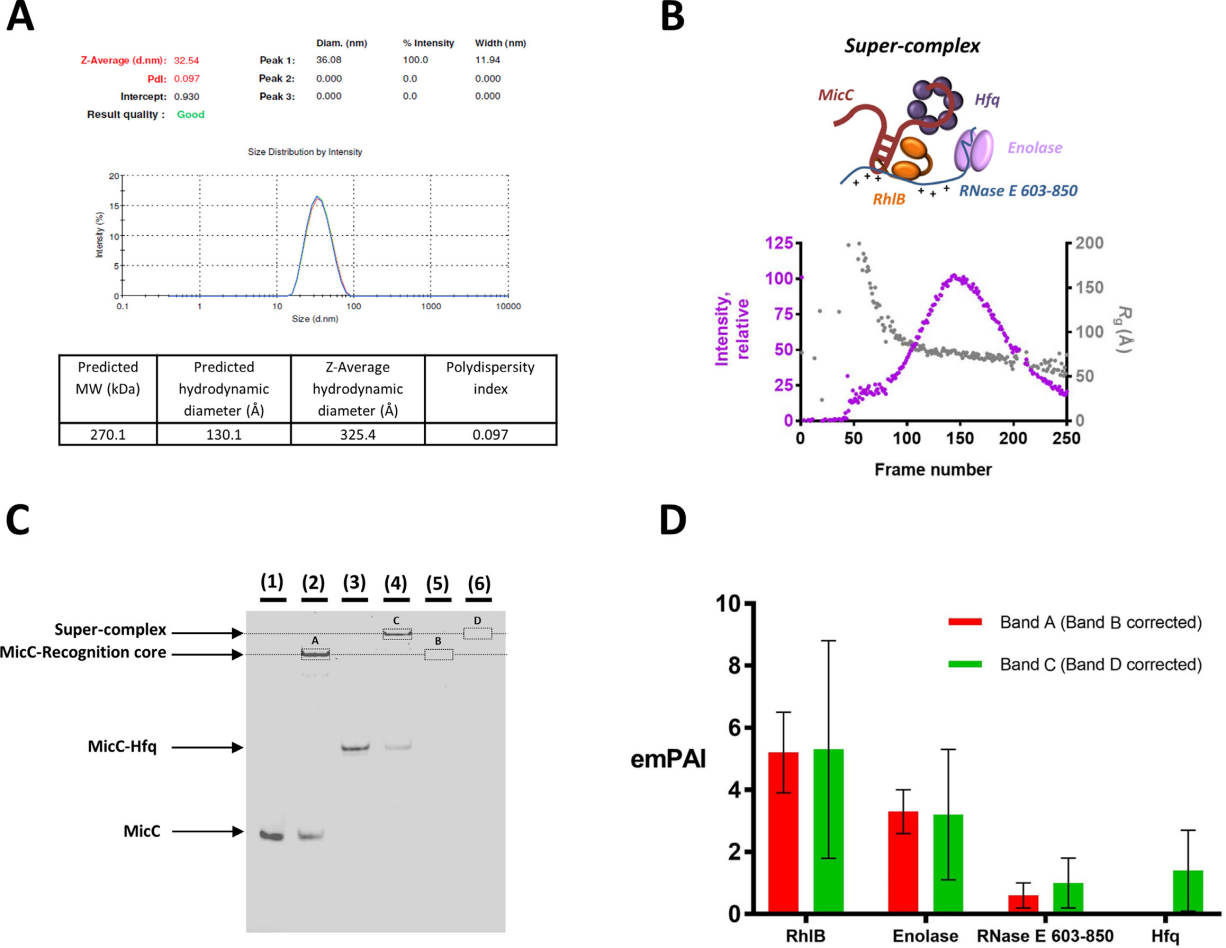
Interactions of the degradosome recognition core with Hfq and the sRNA MicC. (**A**) DLS measurements for the RNase E 603–850/RhlB/enolase/MicC/Hfq ‘super-complex’. Three measurements were carried out. The predicted *M*_w_ is based on a 1:1:1 assembly of the ternary complex: MicC: Hfq. The predicted hydrodynamic diameter is the expected value for a globular particle with the same *M*_w_. The z-average hydrodynamic diameter is the experimental value determined from the DLS measurement and analysis. The polydispersity is <0.2 indicating that the system is monodisperse. (**B**) SEC-SAXS data for the RNase E 603–850/RhlB/enolase/MicC/Hfq ‘super-complex’. The X-ray scattering intensity as a function of elution time (exposure frame) from the size exclusion chromatography column (purple). The Guinier derived radius of gyration (*R*_g_) value for each frame is plotted with elution time (gray). The *R*_g_ deviated significantly from beginning to the end of the peak, indicating high sample heterogeneity. (**C**) Polyacrylamide native electrophoretic mobility shift assay of MicC, recognition core (RNase E 603–850/RhlB/enolase) and Hfq interactions, stained for nucleic acid. MicC (alone in lane 1) interacts with the recognition core (lane 2) and Hfq (lane 3). Upon addition of all three components—recognition core, MicC and Hfq—a super-complex forms (lane 4). In lanes 5 and 6, the recognition core with and without Hfq, respectively, were loaded as controls. (**D**) The emPAI values (49) for bands A and B, corrected for bands C and D, respectively.

The C-terminal region of RNase E has been shown to facilitate the action of the sRNA MicC to trigger degradation of the target mRNA *ompD* (11,33). An interaction between the RNase E recognition core and Hfq was investigated using size-exclusion chromatography, in the presence and absence of the sRNA MicC (Supplementary Figure S8A). A direct interaction between the recognition core and Hfq was not observed. Probing the interaction between the recognition core and Hfq using gel electrophoresis under native conditions also supports this finding (Supplementary Figure S8B). In the presence of MicC however, a super-complex forms, revealing that the interaction is sRNA mediated. While DLS measurements indicate that the particle is non-globular in shape, SEC-SAXS studies reveal that the assembly is highly heterogeneous in conformation, with the *R*_g_ varying greatly across the eluting peak (Figure 6A and B, respectively). Thus, the MicC/Hfq complex may be recognized in multiple ways by the recognition core, indicating that this region of the degradosome has great plasticity.

The above experiments suggest that Hfq does not interact directly with the recognition core region in the degradosome, while the MicC/Hfq complex can. It is possible that the sRNA might be forming a bridging interaction to link the ternary complex and Hfq. Supporting evidence for a direct interaction between MicC and the recognition core is provided by native gel analysis (Figure 6) and corroborated by nuclear magnetic resonance spectroscopy (Supplementary Figure S9). Resonances from the large, slowly tumbling RhlB and enolase polypeptides are probably too broad and weak to detect, so the limited number of intense narrow signals that dominate the one dimensional ^1^H spectrum of the RNase E 603–850/RhlB/enolase complex most likely reflect a highly flexible unstructured portion in RNase E 603–850. In the presence of MicC, the most significant difference is a reduction in intensity for a cluster of sharp resonances at 2.7 ppm. This change suggests that one or more dynamic side-chain sites in RNase E 603–850 interact with MicC, allowing the slowly tumbling sRNA to act as a relaxation sink such that the signals from bound sites become broad and weak. In this way, flexible regions in the recognition core could become conformationally restrained upon binding RNA.

To identify which protein components form part of the recognition core/MicC/Hfq assembly, a native gel shift assay was carried out followed by mass spectrometry analysis. The regions corresponding to shifted species highlighted in the native gel in Figure 6C were excised and analyzed. Using the emPAI (49), which gives an estimate of the abundance relationship between proteins in a sample, the levels of each protein in the assembly were scored (Figure 6D). Regions in the corresponding migration positions in protein only control lanes were also excised and used for background correction. As expected, band A in lane 2 corresponding to a recognition core/MicC shifted species contains all three protein components: RNase E 603–850, RhlB and enolase. The super-shifted species in band B found in the recognition core + MicC + Hfq lane 4 contains all three components of the recognition core at similar levels to those found in band A, in addition to Hfq. This finding suggests that binding of Hfq to this region of the degradosome via RNA does not displace RhlB or enolase.

## DISCUSSION

**Figure 7.**
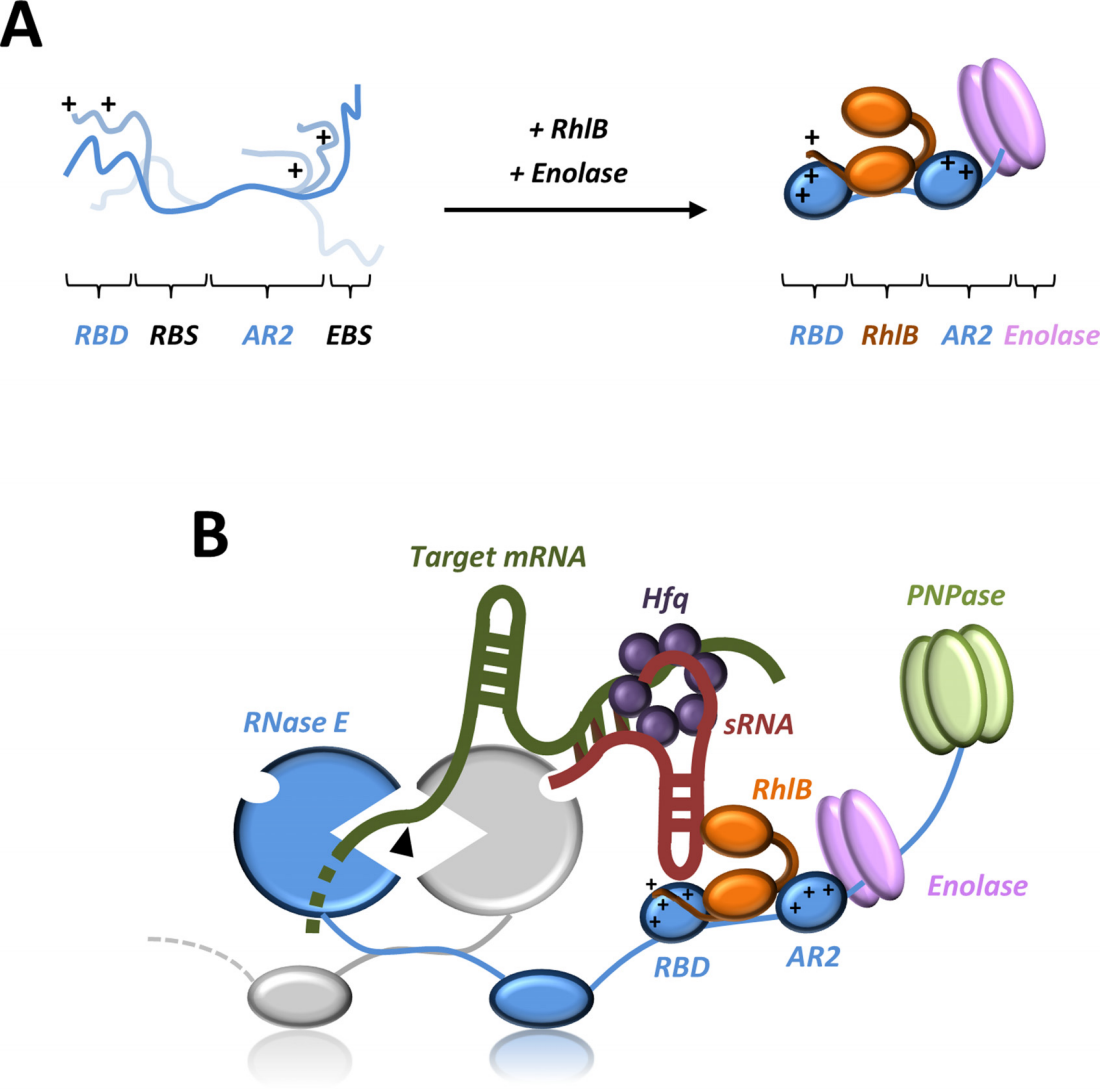
Models for recognition core assembly and sRNA-mediated degradation of mRNA in the RNA degradosome. (**A**) RNase E 603–850 alone is highly flexible in solution, but with a preference for forming conformations which are more extended. Upon addition of RhlB and enolase, the RBD and AR2 form more limited conformations which helps pre-organize these regions for RNA binding protein and/or RNA–protein complexes. (**B**) A possible model for sRNA-induced degradation of target mRNA by the RNA degradosome. A sRNA–Hfq complex interacts within the recognition core region in the CTD of RNase E, via the sRNA, without displacing RhlB and enolase. Although one possible mode of interaction between the sRNA/Hfq/mRNA complex and the recognition core region in the degradosome is depicted, and we anticipate that there are various stages of interaction and remodeling in the process of sRNA-mediated degradation of target mRNA. In this case, the 5′ monophosphate end of the sRNA activates the RNase E catalytic domain by binding in the 5′ sensor domain, rendering the target mRNA susceptible to cleavage as proposed by Bandyra *et al.*, 2012 (11).

Computational analyses predict that the C-terminal domain of RNase E is mostly unstructured, although punctuated by small segments having structural propensity and corresponding to recognition sites for enolase, PNPase and the cytoplasmic membrane, as well as the RBD (15) (Figure 1). In this study, solution data and native mass spectrometry measurements confirm that RNase E 603–850 in isolation is highly dynamic and exhibits physical properties expected for a natively disordered protein. However, upon interaction with RhlB, RNase E 603–850 gains some structural order as indicated by the physical parameters obtained from SAXS and native mass spectrometry measurements. As the RBD region is predicted to have a propensity for forming a coiled coil (15) it might be helped in assuming this conformation when RhlB binds to the adjacent site. Interestingly, the HDX-MS analysis revealed that the solvent accessibility was reduced in the AR2 domain within RNase E 603–850 upon RhlB binding, suggesting that the interaction may induce structural changes in the AR2 region. Furthermore, we find that the interaction between enolase and the RNase E EBS impacts favorably on the RNA-binding affinity of the AR2 region. Taken together, these results lead us to propose a structural model for an RNA/protein recognition core in the C-terminal domain of RNase E (Figure 7A). In this model, the binding of RhlB and enolase facilitates their partner RNase E 603–850 to transition from a highly dynamic state to a molten globule with transient secondary structure elements (36,61). The neighboring RBD and AR2 regions are not expected to form completely rigid structures, but instead are hypothesized to sample a more limited conformational space in which certain residues and motifs are presented for RNA recognition. The dynamic nature of the RBD and AR2 regions is expected to enable the recognition core to have conformability for binding RNA substrates of different shapes, sizes and sequence.

RNase E is membrane-associated *in vivo* and generates transient foci that form on transcripts (17). These foci may be cooperative degradation centers formed by several degradosome particles, and they share remarkable similarities and functional analogy with the eukaryotic ribonucleoprotein (RNP) granules formed by RNA binding and processing enzymes. On the latter, these eukaryotic RNP granules are microscopic structures resembling phase-separated droplets and are proposed to act as ‘nano-organelles’ that are partitioned from the cytoplasm without the requirement for a lipid membrane (61). The liquid–liquid phase separation is postulated to be mediated by disordered regions of RNA-binding proteins that can form new interactions within such droplets. This phasing not only brings about compartmentalization of the enzymes and RNA-binding proteins, but also influences their specificities for nucleic acids. A similar role of the natively unstructured matrix in the channels of nuclear pores has been proposed to underlie the selectivity for transport cargo (62). In the context of the degradosome, extensive disordered regions in the C-terminal tail of RNase E could promote phase separation through self-interaction or distributed contacts with RNA and association with unstructured regions of other degradosome components. Thus, clustering of the ribonuclease on the cytoplasmic membrane may be a 2D analog of the phase transition behavior proposed for RNP granules and could yield highly cooperative behavior of enzyme activities on a bound substrate. The recognition core (Figure 7A) has great plasticity to accommodate numerous RNA species of different sequence and structure, and could contribute to the formation of such proposed RNP granule-like foci.

RNase E is important for the sRNA-mediated degradation of cognate mRNAs (22,33) and a specialized sRNA-Hfq-RNase E complex assembly has been proposed to exist, which is distinct from the ‘typical’ RNA degradosome with its canonical components (23,26). In the model, the RNA chaperone protein Hfq forms a direct interaction with the recognition core region of RNase E, connecting it to a particular sRNA and displacing RhlB and enolase which normally bind there (26,50). However, a subsequent study showed that a direct interaction between Hfq and RNase E (628–843) does not occur in absence of RNA (47). Here we have used several *in vitro* techniques to characterize the interaction between the degradosome recognition core and a sRNA–Hfq complex. In our experiments, the sRNA MicC is clearly required to mediate the interaction between Hfq and the recognition core, indicating that a direct protein–protein interaction is unlikely to occur *in vivo*. Furthermore, interaction between the recognition core and the MicC–Hfq complex does not displace RhlB or enolase, suggesting that both may play important roles in sRNA–mRNA riboregulation by RNase E. Our findings differ from earlier reports, in which sRNA–Hfq interaction was thought to displace the helicase and enolase from the degradosome in co-immunoprecipitation experiments (26,59). Indeed, it is interesting to note that a recent study revealed that in *E. coli* cells exposed to anaerobic conditions, the specific disruption of enolase-RNase E interaction leads to the destabilization of the sRNA DicF and higher levels of its target mRNA FtsZ, resulting in abnormal cell morphology (63). Based on our observations, we suggest that enolase bound within the degradosome could be involved in the stabilization of DicF by inducing a structural conformation in the AR2 RBD of RNase E which enables this region to more favorably interact with the sRNA and confer to it some protection.

We have devised an alternative model for sRNA-mediated degradation of target mRNAs by the degradosome to that which was previously suggested (26), whereby an sRNA–Hfq complex interacts within the RNase E 603–850 region, without displacing the canonical components enolase and RhlB found there (Figure 7B). In our model, the interaction between the degradosome and Hfq is mediated by the sRNA, which also activates the catalytic activity of RNase E through the 5′ monophosphate moiety binding to the 5′ sensor sub-domain in the N-terminal domain of the ribonuclease, priming it for the endonucleolytic cleavage of a complementary mRNA (11). For sRNAs that do not act in a 5′ monophosphate dependent pathway, the activation of RNase E might be mediated through recognition of RNA structural signatures presented *in trans* by the sRNA, in analogy to the proposed mechanism for the 5′ end bypass pathway of certain RNA substrates by the enzyme (64). The recognition core region of the degradosome could confer some protection to the sRNA until the complementary mRNA binds and the opportune moment occurs for coupled or sequential degradation of the sRNA–mRNA pair. The sRNA–mRNA pairing is facilitated by Hfq, and this chaperone is likely to dissociate at some point before or during the RNA degradation process. The RBD and AR2 domains flanking the enolase and RBSs in RNase E are envisaged to contribute to binding the sRNA, the mRNA or the sRNA–mRNA pair, and the physical presence of enolase and RhlB in the recognition core region are expected to enhance this interaction and may be important for the displacement of Hfq upon remodeling of the complex.

This model bears some functional analogy to a transient ‘Amplifier’ complex identified in the germline cells of the insect *Bombyx mori* BMN4 (65). In perinuclear cytoplasmic granules, the DEAD box helicase VASA provides a loading platform for the PIWI-endonucleases Siwi and Ago3—involved in processing secondary piRNAs (PIWI RNAs) from transposable element transcripts. In the Amplifier complex, VASA acts like a clamp by holding onto the precursor sense piRNA and protecting it from endoribonucleolytic cleavage by Siwi. It is possible that RhlB carries out a similar role in the protection of sRNA in complex with Hfq from cleavage by RNase E or PNPase, until the complementary target mRNA presents itself. The open conformation of the helicase could then result in sRNA release, exposing it to RNase E activity. This hypothesis awaits further experimental evaluation.

## DATA ACCESS

The coordinates and structure factors have been deposited with the PDB ID: 5OHG. SAXS data has been deposited at SASDB under accession codes SASDCY4, SASDCZ4, SASDC25.

## Supplementary Material

Supplementary Data
