# Supplementary material for: Analysis of the natively unstructured RNA/protein-recognition core in the Escherichia coli RNA degradosome and its interactions with regulatory RNA/Hfq complexes
Source: Nucleic Acids Res. 2017 Nov 9;46(1):387–402. doi: 10.1093/nar/gkx1083 (PMC5758883; doi:10.1093/nar/gkx1083)
Supplement: Supplementary Data [file gkx1083_supp.pdf]

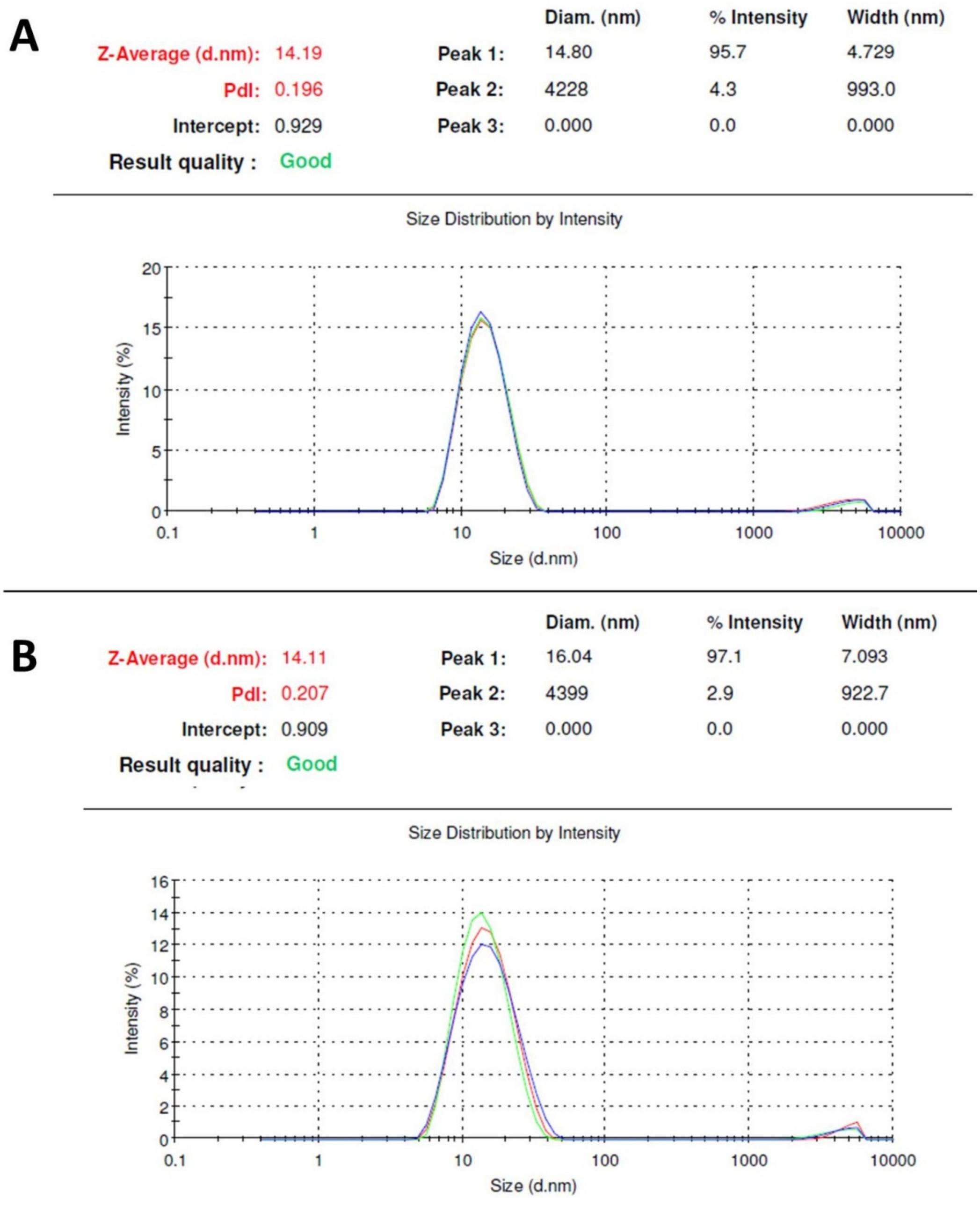

Sample	Predicted MW (kDa)	Predicted hydrodynamic diameter (Å)	Z-Average hydrodynamic diameter (Å)	Polydispersity index
Ternary complex	168.5	106.1	141.9	0.196
Binary complex	77.2	76.0	141.1	0.207

Supplementary Figure 1. Dynamic Light Scattering (DLS) measurements for degradosome subassemblies. Three measurements were carried out for each sample. **(A)** The RNase E 603-850/RhlB/enolase ‘ternary complex’. **(B)** The RNase E 603-850/RhlB ‘binary complex’. Predicted

molecular weights shown in the table (inset), are based on a 1:2:1 complex assembly of RNase E 603-850: enolase: RhlB and a 1:1 assembly of RNase E 603-850: RhlB for the ternary complex and binary complex, respectively. The predicted hydrodynamic diameter is that expected for a globular particle of the same molecular weight. The z-average hydrodynamic diameter is the experimental value determined from the DLS measurement and analysis. The polydispersity should be 0.2 or less to indicate that the system is monodisperse.

DLS Methods: Samples of RNase E 603-850/RhlB/enolase ternary complex and RNase E 603-850/RhlB binary complex, at 3.1 mg/ml and 1.3 mg/ml, respectively, in 50 mM Tris HCl pH 7.5, 100 mM NaCl, 100 mM KCl, 5 mM $MgCl_2$ and 5 mM DTT, were prepared. For each sample, 70 µl was transferred to a quartz cuvette (Sigma Z637939) and three independent DLS measurements were carried out at 25 °C, using a Malvern Zetasizer NanoS instrument, detecting scattered light at an angle of 173°.

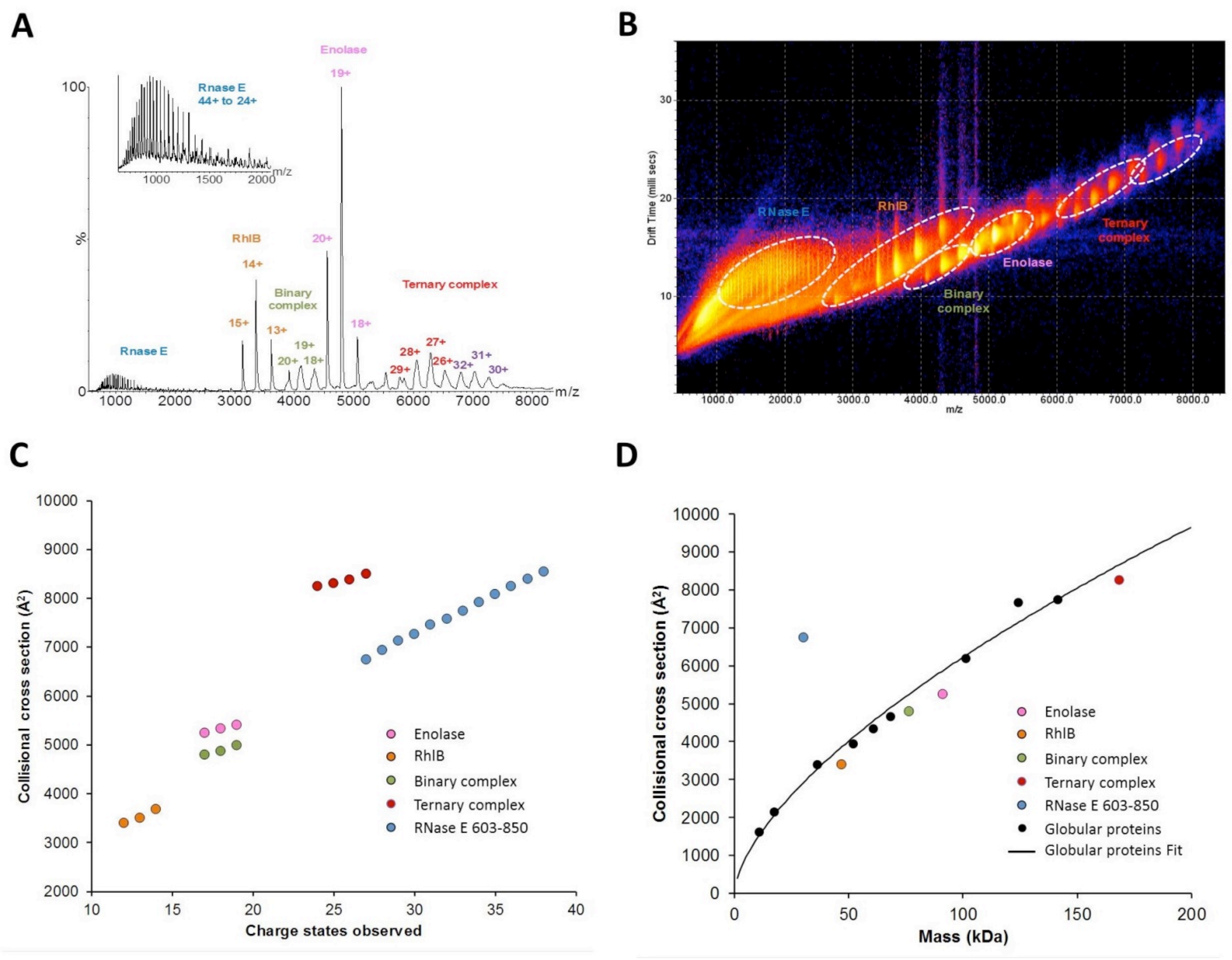

Complex	Expected MW (Da)	MW from native MS (Da)	Collisional cross section (Å^2)
**RNase E 603-850**	30,127.1	30,056 ± 9	6741.9
**RhlB**	47,125.9	47,016 ± 4	3401.4
**Enolase**	91,291.8	91,067 ± 7	5244.8
**Binary complex**	77,235.0	78, 454 ± 50	4792.1
**Ternary complex**	168,508.5	169,651 ± 40	8250.6

Supplementary Figure 2. Ion mobility mass spectrometry analysis of the RNase E 603-850/RhlB/enolase ternary complex. **(A)** Native mass spectra of ternary complex in 250 mM ammonium acetate pH 7.0. Inset plot shows the RNase E 603-850 charge states in more detail. **(B)** DriftScope plot showing the drift times of the species detected in the ternary complex. **(C)** Drift times for the ternary complex and constituents extracted from DriftScope were converted to collisional cross section (CCS) values using a calibration curve generated by measuring standard proteins of known CCS. Here the CCS values are plotted with charge state. **(D)** Estimated CCS values for the species observed in the ternary complex compared to globular proteins. The lowest charge

state for each protein/complex was plotted against its molecular mass and this is compared to standard globular proteins. The molecular masses and collisional cross sections measured from native MS and ion mobility-mass spectrometry for the RNase E 603-850/RhlB/enolase ternary complex and its constituents are presented in the table (inset). Larger errors in MW for the ternary and binary complexes are due to broad peaks that are typically observed for native MS. Collisional cross sections for the lowest charge state (most native-like) are also tabulated.

Native mass spectrometry experiments were carried out on a Waters Micromass QTOF2 modified for high mass complexes. Protein complexes were buffer exchanged into 250 mM aqueous ammonium acetate (pH 7.0) using Micro Bio-Spin P-6 columns (Bio-Rad), at concentrations of 10 µM. Aliquots of 3-5 µL were transferred to gold-coated borosilicate needles prepared in-house. Samples were ionised with a capillary voltage of 1.4 V and a cone voltage of 40 V with the nanaflow gas maintained at 0.5 bar. The source temperature was 80 °C and the collision energy was 10 V. TWIM-MS experiments were conducted on a Synapt HDMS G2 instrument (Waters, Milford, MA, USA). The instrument was tuned to ensure the preservation of non-covalent interactions and for high mass complexes, using the following parameters: capillary 1.3 kV; sample cone 40 V; source temperature 30 °C; extraction cone 0.5 V; nanoflow gas pressure 0.1 bar; trap collision energy 10 V; transfer collision energy 10 V; backing pressure 4.7 mbar, trap pressure 3.5 mbar. Gas pressure in the ion-mobility cell was 3.0 mbar, He and N_2 gas flows 180 ml/min and 90 ml/min respectively with a trap bias of 50 V. The travelling wave velocity was 800 m/s with a travelling wave height of 40 V. The data were acquired and processed with MassLynx v4.1 software (Waters), and drift times extracted using Driftscope v2.5 (Waters). The experimental collision cross sections (CCS) of the protein complexes were determined by calibration with known protein cross-sections determined under native conditions as described previously.

Ion mobility spectrometry–mass spectrometry (IMS–MS) Synapt HD mass spectrometer was used to perform IMS–MS measurements for the ternary complex. The complex was exchanged into 0.20 M ammonium acetate pH 87.0. The ion–mobility cell was filled with nitrogen gas at 0.5 mbar pressure and the traveling wave velocity was 250 ms−1. Five wave heights (7.0, 8.0, 9.0, 10.0 and 11.0 V) were used for measurements. The reported collision cross section (CCS) values were an average of the data recorded over all of the wave heights. CCS calibration was performed using exactly identical conditions described above on four proteins (alcohol dehydrogenase, concanavalin A, pyruvate kinase and avidin) and involved validation through comparison with CCS data from literature. The drift times for all charge states were obtained using Micromass MassLynx 4.1 and DriftScope (Waters). Theoretical CCS values from PDB files were calculated using the CCScalc software (Waters), which utilizes a projection approximation (PA) algorithm.

Supplementary Table 1. SAXS data collection and structural parameters

	**RNase E 603-850**	**Binary complex**	**Ternary complex**	**Super-complex**
**Data collection parameters**				
Beamline	Swing, Soleil			
Wavelength (Å)	1.022			
q range ($Å^{-1}$) *	0.0062-0.6142			
Temperature (K)	288			
Conc (mg/ml)/ Vol (μl)	12.0/50	22.3/50	9.1/50	10.2/50
**Structural parameters**				
I(0) (relative) (from *P(r)*)	0.05 ± 0.01	0.22 ± 0.01	0.19 ± 0.01	-
R_g (Å) (from *P(r)*)	57.42 ± 0.14	58.75 ± 0.15	69.71 ± 0.19	-
I(0) (cm^{-1}) (from Guinier)	0.05 ± 0.01	0.22 ± 0.01	0.19 ± 0.01	-
R_g (Å) (from Guinier)	52.55 ± 0.31	53.75 ± 0.71	63.90 ± 1.28	-
D_{max} (Å)	275 ± 10	295 ± 10	305 ± 10	-
Porod volume V_p ($Å^3$)	139,000	183,000	280,000	-
Excluded volume V_{ex} ($Å^3$) (*DAMMIN*/P1)	106,400 ± 2,100	252,700 ± 1,900	390,000 ± 3,200	-
**Molecular mass determination (Da)**				
From Porod volume (V_p/1.7)	81,800	107,700	165,000	-
From excluded volume (V_{ex}/1.7)	62,600 ± 1,200	148,700 ± 1,100	231,800 ± 1,900	-
From SAXS MoW2 / ($q$max, $Å^{-1}$)	48,700 / 0.39	139,000 / 0.18	208,500 / 0.15	-
From Sequence (including hexa-histidine tag)	30,127	77,235	168,509	-
**Modelling parameters**				
Shape reconstruction	*DAMMIN*	*GASBOR*	*GASBOR*	-
Symmetry	P1	P1	P1	-
NSD (var) / # of models	0.83 (0.03) / 10	2.04 (0.09) / 10	2.40 (0.10) / 10	-
χ^2 range	23.39 - 184.39	16.39 - 46.72	4.97 - 8.65	-
Flexibility assessment χ^2 range / # runs	*EOM/FULCHER* 5.3 / 10	- -	- -	- -
**SAXSDB codes**	SASDCY4	SASDCZ4	SASDC25	-
**Software employed**				
Primary data reduction	*FOXTROT*			
Data processing	*DATASW, PRIMUS, GNOM*			
Computation of component model intensities	*CRYSOL*			
Model representations	*PYMOL, CHIMERA*			

Abbreviations: *I(0)*, extrapolated scattering intensity at zero angle; R_g, radius of gyration calculated using either Guinier approximation (from Guinier plot) or the indirect Fourier transform package GNOM [from *p(r)*]; M_r, molecular mass; D_{max}, maximal particle dimension; V_p, Porod volume; V_{ex}, particle excluded volume. '-': analysis not performed.

*Momentum transfer $|q| = 4\pi\sin(\theta)/\lambda$.

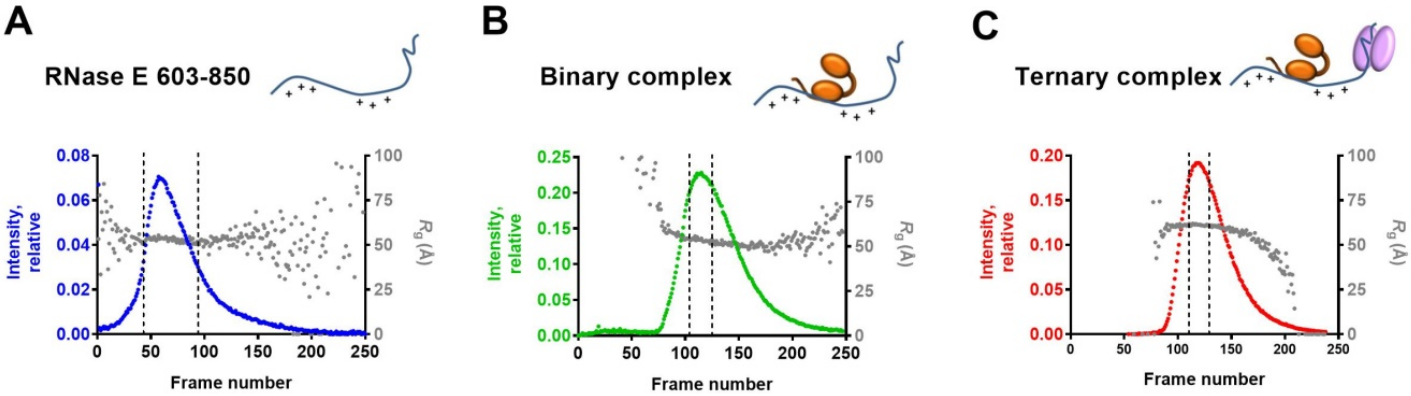

Sample	Frame range	Average R_g (Å) ± st. dev.
RNase E 603-850	38-98	51.72 ± 1.12
Binary complex	105-125	53.18 ± 0.95
Ternary complex	110-130	62.36 ± 0.99

Supplementary Figure 3. Analysis of SEC-SAXS data for degradosome subassemblies. The X-ray scattering intensity as a function of elution time (exposure frame) from the size exclusion chromatography column for **(A)** RNase E 603-850, **(B)** the RNase E 603-850/RhlB binary complex and **(C)** the RNase E 603-850/RhlB/enolase ternary complex. The Guinier derived radius of gyration (R_g) value for each frame is plotted with elution time. The average R_g within the selected range for subsequent analysis (vertical dotted line range) with respective standard deviation for RNase E 603-850, the binary complex and the ternary complex in the table (inset).

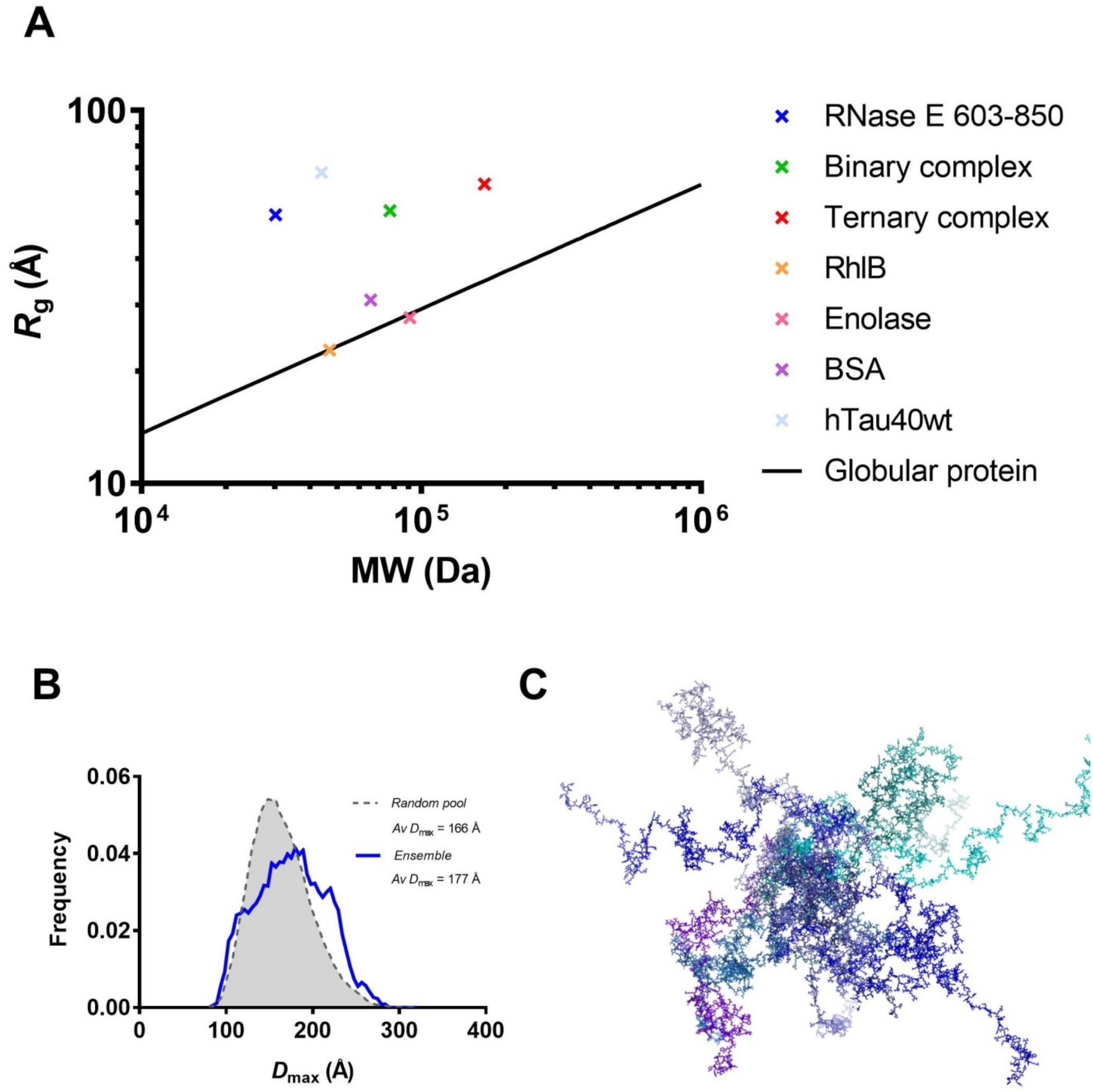

Model	R_g (Å)	D_{max} (Å)
Smallest	35.67	135.70
Largest	84.16	260.90
Ensemble	56.09	190.19

Supplementary Figure 4. Flexibility analysis of degradosome subassemblies. (A) The expected R_g for a globular protein plotted as a function of molecular weight using the following equation from Putnam et al., 2007: R_g (globular protein) = $(3/5)^{1/2}$ $[(\text{MW (Da)}/0.44\ (\text{Da/ Å}^3) * (3/4\pi)]^{1/3}$(1). RNase E 603-850 (blue), binary complex (green) and ternary complex (red) deviate from the relationship observed for globular proteins (black line), such as enolase (pink) and RhlB (orange). R_g values for enolase and RhlB were determined using the crystal structure of enolase (PDB: 3H8A) and a homology model of the helicase in the program CRYSOL (2). The R_g values for BSA (purple) and hTau40wt (light blue), globular and highly flexible proteins, respectively, are also included for comparison (3). **(B)** D_{max} distribution of RNase E 603-850 (related to Figure 3D and E) ensemble

generated in EOM (4). **(C)** All-atom models of RNase E 603-850 generated by EOM/FULCHER (Shkumatov A.V., unpublished data) which as an ensemble give a good fit to the scattering intensity (see Figure 3E). Presented in the table (inset) are the R_g and D_{max} values for the smallest and largest conformation, in addition to the average values for the ensemble.

A

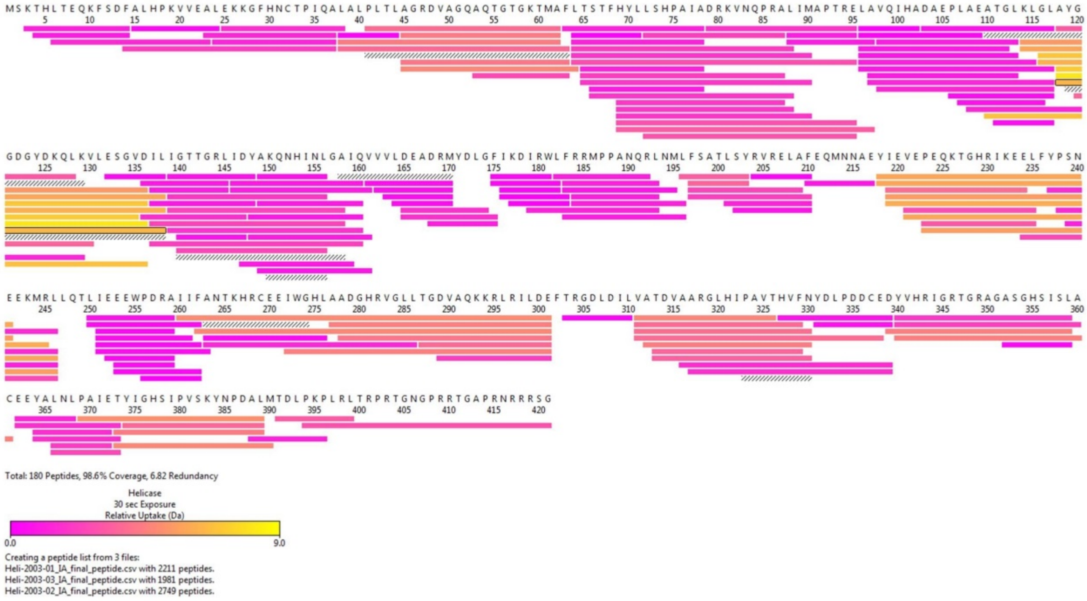
Total: 180 Peptides, 98.6% Coverage, 6.82 Redundancy
Helicase
30 sec Exposure
Relative Uptake (Da)
0.0
9.0
Creating a peptide list from 3 files:
Heli-2003-01_IA_final_peptide.csv with 2211 peptides.
Heli-2003-03_IA_final_peptide.csv with 1981 peptides.
Heli-2003-02_IA_final_peptide.csv with 2749 peptides.

B

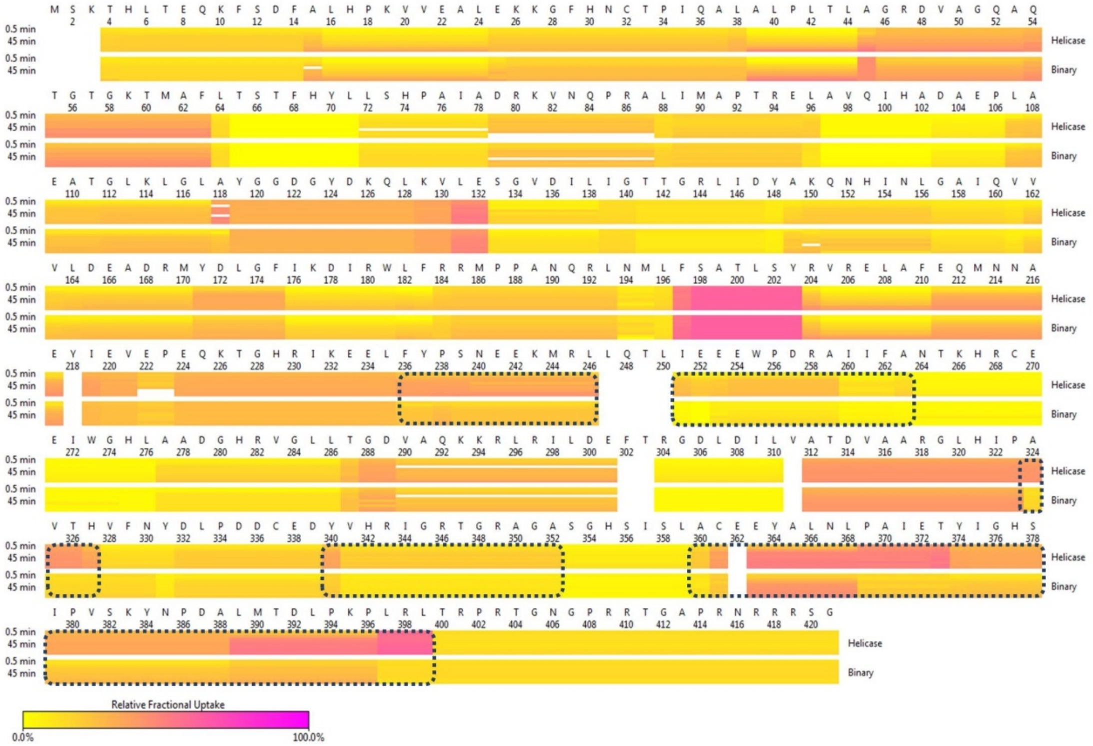
0.5 min
45 min
Helicase
Binary
Relative Fractional Uptake
0.0%
100.0%

C

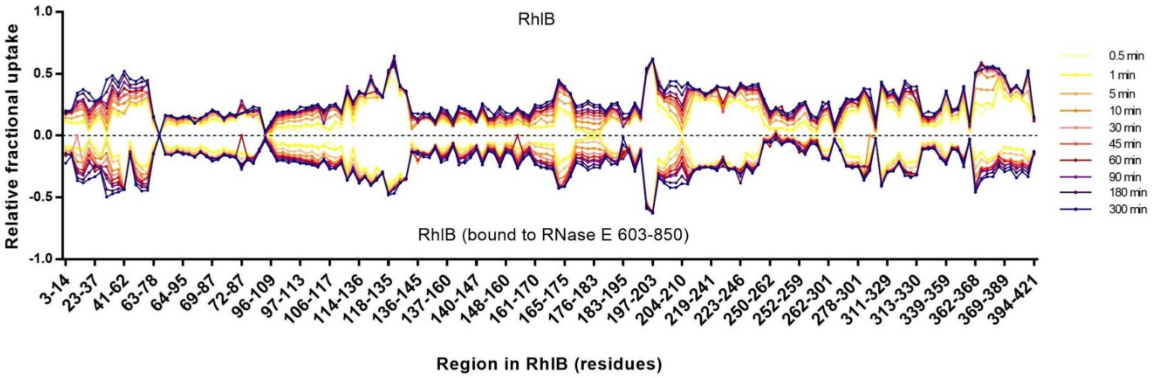

D

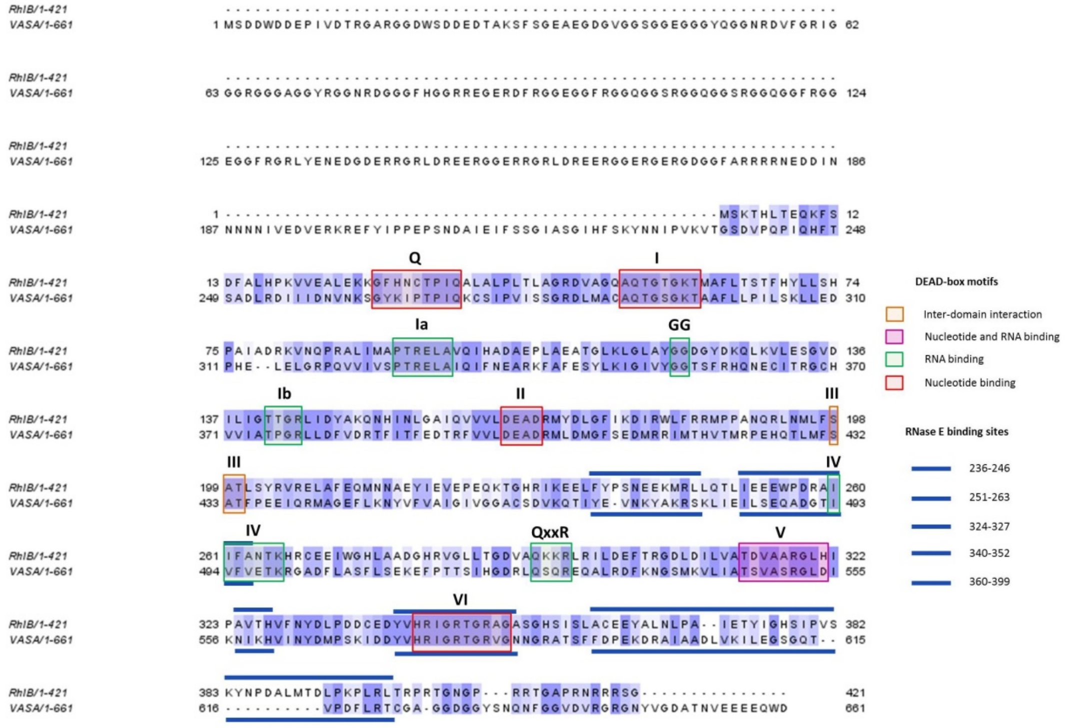

Supplementary Figure 5. Hydrogen/deuterium exchange coupled to mass spectrometry (HDX-MS) mapping of the RNase E interaction site in RhlB. (A) Peptide coverage map of RhlB, displaying coverage of 98% across the entire sequence length. **(B)** RhlB heat map revealing the relative change in H/D exchange throughout the sequence. (Top) RhlB alone. (Bottom) RhlB bound to RNase E 603-850. Exposure times 0.5 and 45 seconds are shown for comparison. **(C)** Butterfly plot to show the change in H/D exchange with exposure time, across the sequence length. (Top) RhlB alone. (Bottom) RhlB in complex with RNase E 603-850. **(D)** Sequence alignment of *Escherichia coli* RhlB (Top) and VASA helicase from *Drosophila melanogaster* (Bottom). Residues are coloured in blue to white from

most conserved to non-conserved. The most highly conserved regions correspond to nucleotide (red), RNA (green), Nucleotide *and* RNA binding (pink) and inter-domain interaction (orange) motifs. The putative RNase E interaction sites in RhlB, as identified by the HDX-MS analysis are indicated by dark blue lines.

A

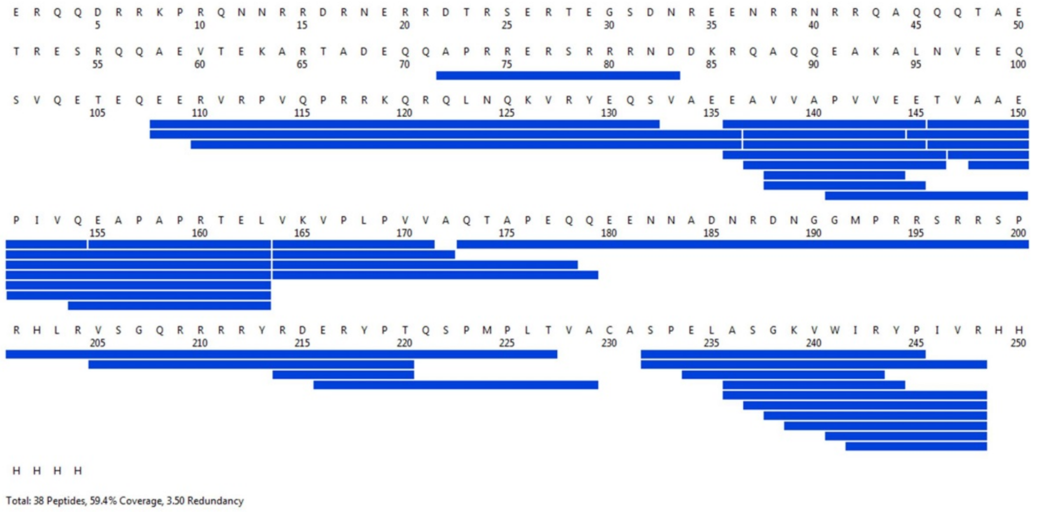
Total: 38 Peptides, 59.4% Coverage, 3.50 Redundancy

B

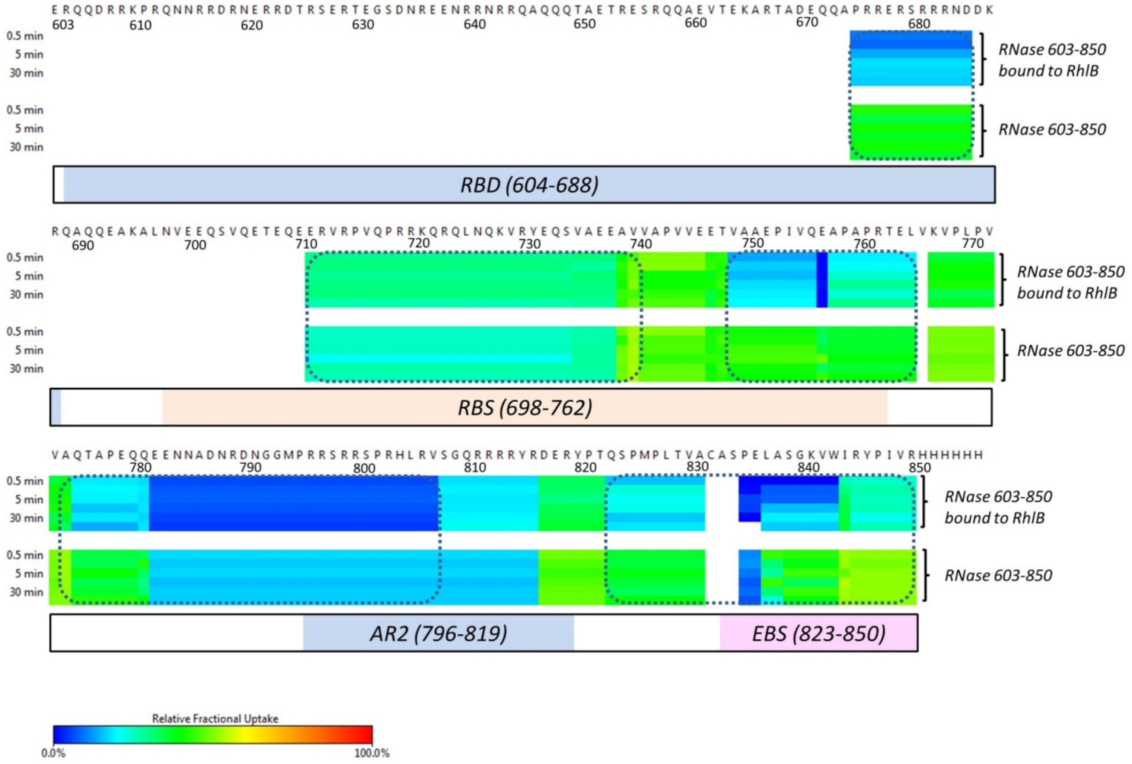
0.5 min
5 min
30 min
RNase 603-850 bound to RhlB
RNase 603-850
RBD (604-688)
RBS (698-762)
AR2 (796-819)
EBS (823-850)
Relative Fractional Uptake
0.0%
100.0%

C

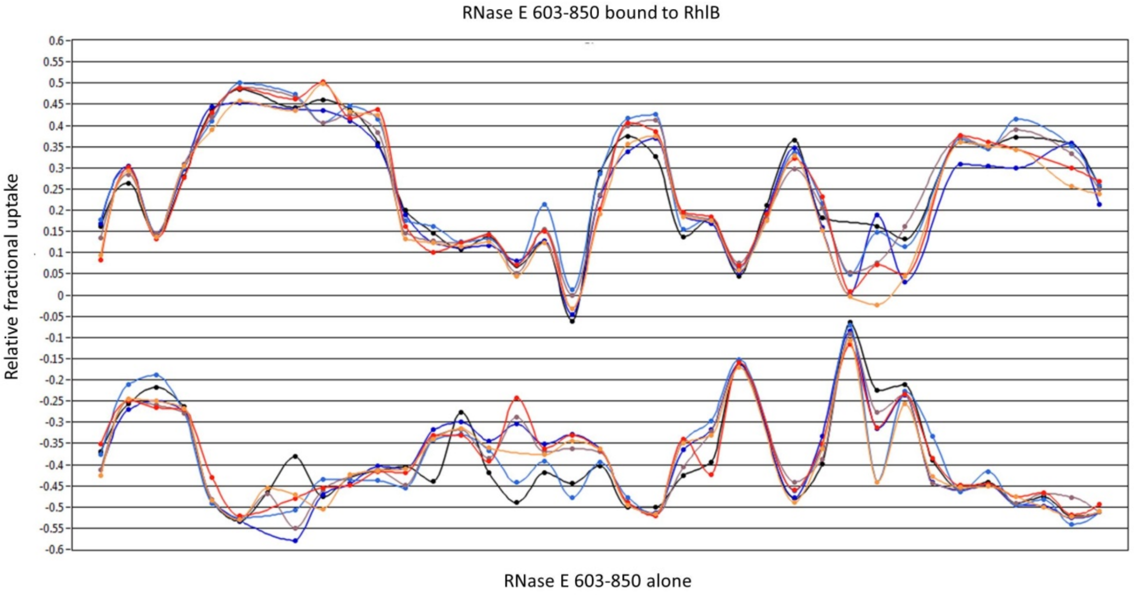

D

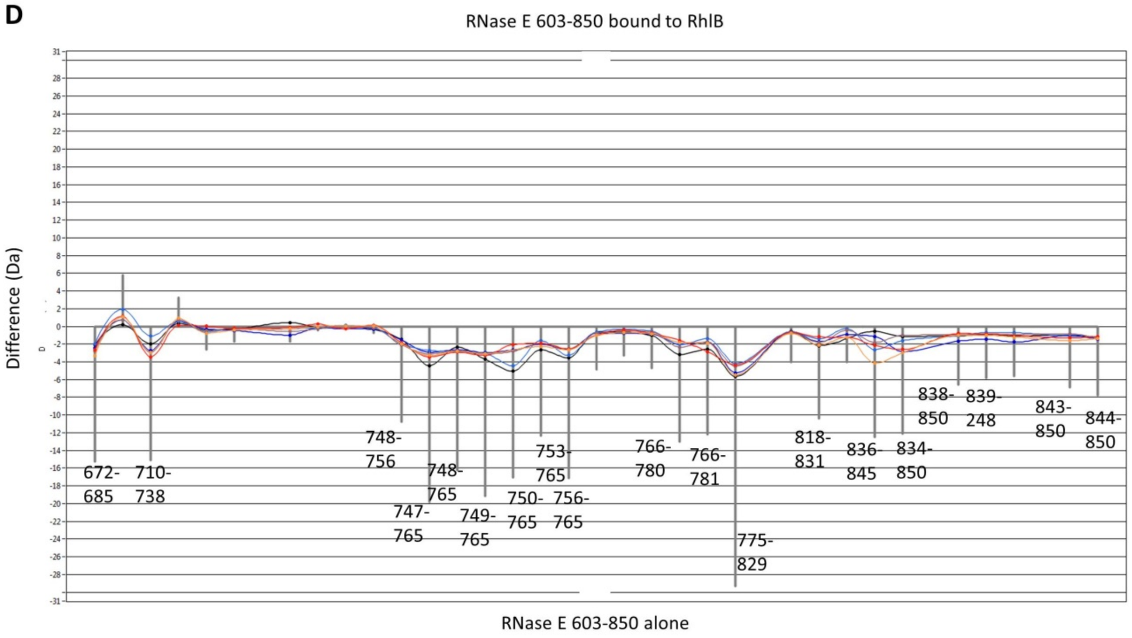

Supplementary Figure 6. HDX-MS analysis of RNase E 603-850 alone and in complex with RhlB. (A) Peptide map of RNase E 603-850, displaying coverage of 59%. Although the peptide coverage in RNase E 603-850 was incomplete due to a limited number of pepsin cleavage sites in the N-terminal part of the protein, regions corresponding to residues 675-685 and 711-850 were detected in the mass spectrometer. **(B)** Heat map of RNase E 603-850 revealing the relative change in H/D exchange throughout the sequence. (Top) RNase E 603-850 in complex with RhlB. (Bottom) RNase E 603-850 alone. For comparison, 0.5, 5 and 30 minutes are shown. **(C)** Butterfly plot of RNase E 603-850 displaying the change in H/D exchange with time. (Top) RNase E 603-850 in complex with RhlB. (Bottom) RNase E 603-850 alone. **(D)** Difference in H/D exchange between RNase E 603-850 alone and bound to RhlB. Alone, RNase E 603-850 has a greater exposure to the solvent indicating that it

samples more open conformation. Overall, RNase E 603-850 is also more open alone than when bound to RhlB, as the solvent accessibility is clearly reduced in many areas upon complex formation.

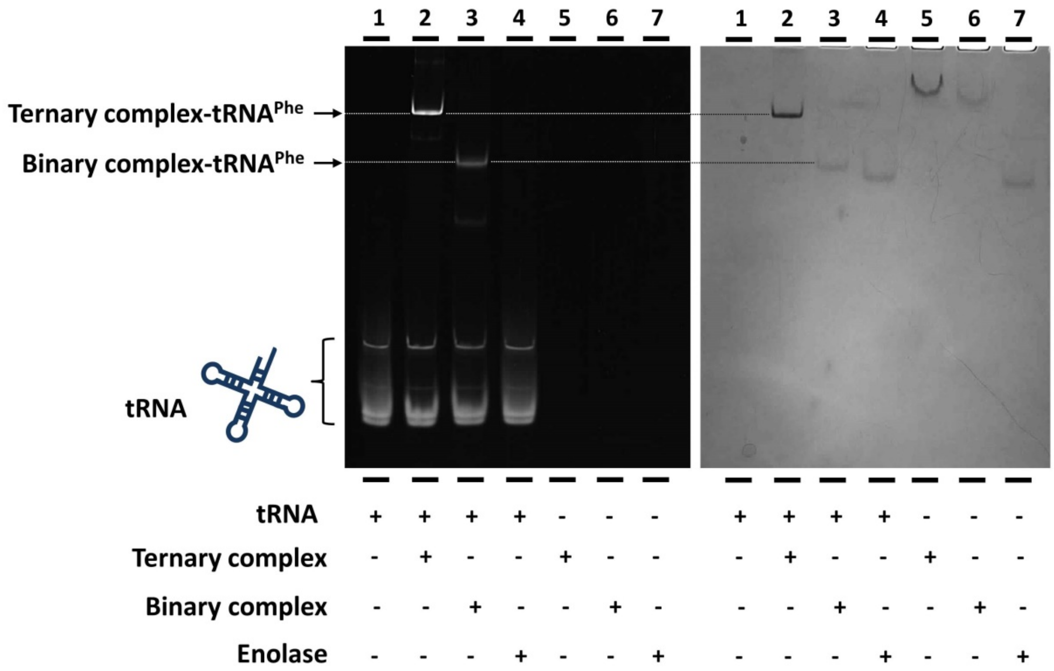

Supplementary Figure 7. Native polyacrylamide gel shift assay probing the interaction of *E. coli* tRNA^{Phe} with the ternary complex (RNase E 603-850-RhlB-enolase), binary complex (RNase E 603-850+RhlB) and enolase. The gel is SYBR Gold stained (left) and coomassie stained (right) for RNA and protein visualisation, respectively. tRNA runs towards the end of the gel (lane 1), and interacts with the ternary complex (lane 2) and the binary complex (lane 3), but not enolase (lane 4). Comparing the two stained versions of the gel, tRNA forms a shifted species with the ternary complex and binary complex, but not enolase. The multiple bands for tRNA alone may represent slightly different structural conformations or charged states in these buffer conditions.

A

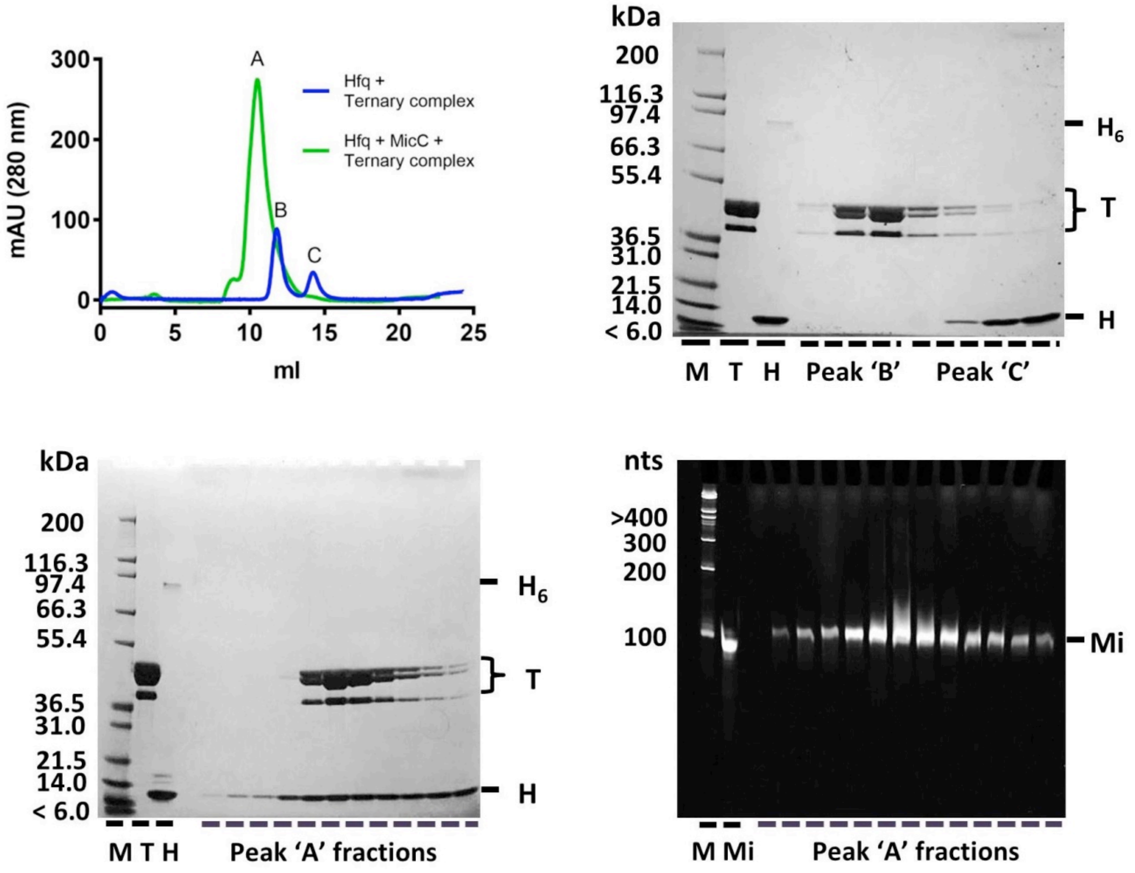

B

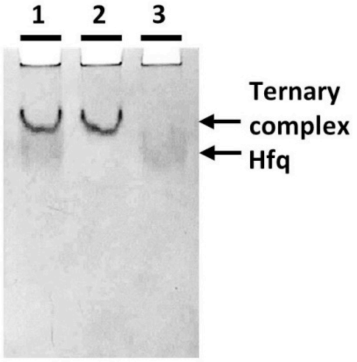

Supplementary Figure 8. The sRNA MicC mediates the interaction between the RNase E 603-850/RhlB/enolase ternary complex and Hfq. (A) SEC profile with 280 nm absorbance measurement of the ternary complex + Hfq (blue) and the ternary complex + MicC + Hfq (green) (Top left). Protein denaturing gel with Ternary complex + Hfq peak fractions (Top right). Protein denaturing gel with

Ternary complex + MicC + Hfq peak fractions (Bottom left). RNA denaturing gel with Ternary complex + MicC + Hfq peak fractions (Bottom right). **(B)** Native gel stained with coomassie indicates that the ternary complex does not interact with Hfq in the absence of RNA. Ternary complex + Hfq (lane 1), ternary complex (lane 2) and Hfq (lane 3).

Samples of the recognition core and Hfq, in the presence and absence of MicC, were prepared by adding each component in equimolar amounts, at a final concentration of 30 µM. 250 µl of each sample was loaded onto a Superdex 200 Increase 10/300 gel filtration column (GE Healthcare), equilibrated with buffer (50 mM Tris-HCl pH 7.5, 250 mM KCl, 250 mM NaCl, 10 mM $MgCl_2$ and 10 mM DTT). Selected fractions from the eluting peaks were then analysed by SDS-PAGE and Urea-PAGE in order to investigate the presence of the protein components and MicC, respectively.

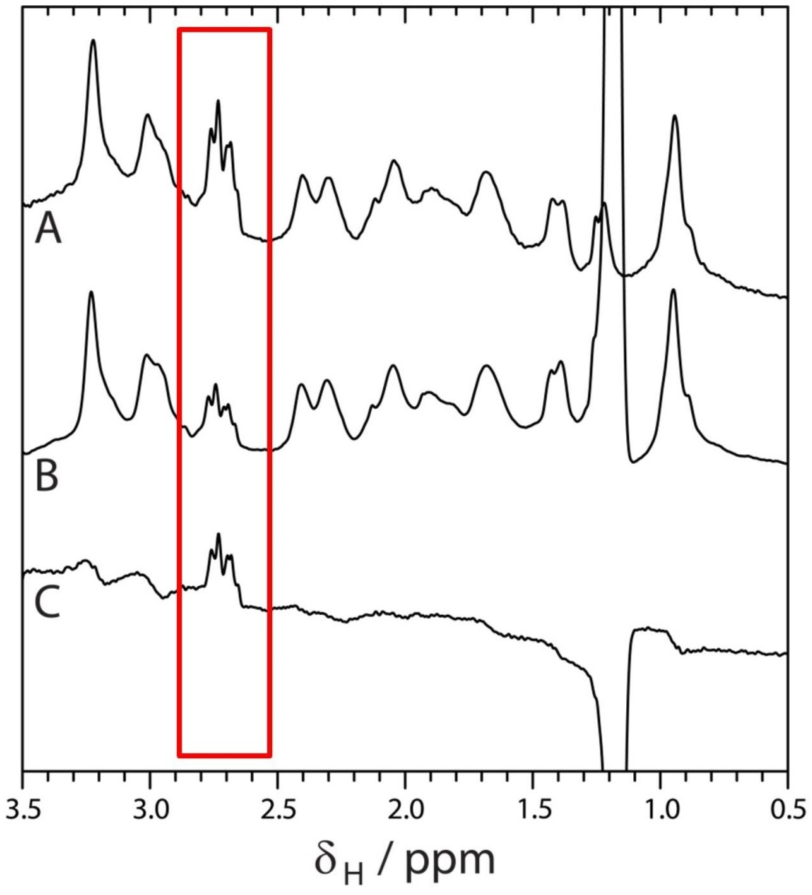

Supplementary Figure 9. NMR spectroscopy of the RNase E 603-850/RhlB/enolase ternary complex in the presence and absence of the sRNA MicC. (A). 1D ^{1}H NMR spectrum of the RNase E 603-850/RhlB/enolase ternary complex. **(B).** ^{1}H spectrum of the ternary complex in the presence of MicC sRNA. **(C).** Difference spectrum (**A** minus **B**). Highlighted in red is the cluster of sharp resonances which display a reduction in intensity upon addition of MicC to the sample.

All samples for NMR spectroscopy were prepared at final concentrations of 300 μM for RNase E 603-850/RhlB/enolase ternary complex and 150 μM MicC in 50 mM Tris HCl pH 7.5, 0.1 M NaCl, 0.1 M KCl and 5 mM $MgCl_2$ supplemented with 10 % D_2O (Sigma) and 0.0025% 3,3,3-trimethylsilylpropionate (Sigma) in 5mm Ultra-Imperial grade NMR tubes (Wilmad). Spectra were recorded at 288 K on a Bruker DRX500 spectrometer equipped with a z-shielded gradient triple resonance probe. 1D 1H spectra were acquired by averaging 32 scans using the first row of a NOESY experiment with Watergate solvent suppression (5).

1. Putnam, C. D., Hammel, M., Hura, G. L., & Tainer, J. A. (2007). X-ray solution scattering (SAXS) combined with crystallography and computation: defingin accurate

macromolecular structures, conformations and assemblies in solution. *Quarterly Reviews of Biophysics, 40(3), 191-285.*

2. Svergun, D., Barberato, C. & Koch, M. H. CRYSOL - A program to evaluate X-ray solution scattering of biological macromolecules from atomic coordinates. *J. Appl. Crystallogr.* **28,** 768–773 (1995).
3. Gorantla, N. V., Shkumatov, A. V. & Chinnathambi, S. in *Methods in Molecular Biology* **1523,** 3–20 (2017).
4. Tria, G., Mertens, H. D. T., Kachala, M. & Svergun, D. I. Advanced ensemble modelling of flexible macromolecules using X-ray solution scattering. *IUCrJ* **2,** 207–217 (2015).
5. Cavanagh, J., Fairbrother, W., Palmer III, A., Rance, M. & Skelton, N. *Protein NMR spectroscopy. Principles and Practice. Elselvier Academic Press* **2nd,** (2007).
